# Efficacy, Safety, and Patient Reported Outcomes of Rhenium-Skin Cancer Therapy for Non-Melanoma Skin Cancer: 1-Year Results from the EPIC-Skin Study

**DOI:** 10.1016/j.adro.2025.101802

**Published:** 2025-04-29

**Authors:** Giuseppe Cardaci, Siddhartha Baxi, Saima Vohra, Cody Allison, Angela Hong, Nicola Mulholland, Mike Sathekge, Kgomotso Mokoala, Martin Heuschkel, Julia Tietze, Siroos Mirzaei, Gerhard Dahlhoff

**Affiliations:** a Department of Nuclear Imaging, Hollywood Private Hospital, Nedlands, Australia; b GenesisCare, John Flynn Hospital, Tugun, Australia; c Avion Medical, Melbourne, Australia; d OncoBeta Therapeutics, Brisbane, Australia; e GenesisCare, North Shore Health Hub, St Leonards, Australia; f Melanoma Institute Australia, Poche Centre, Crows Nest, Australia; g Department of Nuclear Medicine, Kings College Hospital NHS Foundation Trust, Denmark Hill, London, UK; h NuMeRI, University of Pretoria and Steve Biko Academic Hospital; i Department of Nuclear Medicine, University Medical Center Rostock, Rostock, Germany; j Clinic and Polyclinic for Dermatology and Venereology, University Medical Center Rostock, Rostock, Germany; k Clinic Ottakring, Institute of Nuclear Medicine with PET-Center, Vienna, Austria; l OncoBeta GmbH, Munich, Germany

## Abstract

**Purpose:**

Rhenium-skin cancer therapy (SCT) is an innovative, noninvasive radionuclide treatment for nonmelanoma skin cancer (NMSC), which is administered in a single outpatient treatment session. A global, multicenter, single-arm, phase 4 post-marketing clinical study was established to evaluate efficacy, safety, cosmesis, and patient-reported outcomes of OncoBeta rhenium-SCT for NMSC. This report details scheduled 12-month interim results, including toxicity, cosmesis, and patient-reported outcomes.

**Methods and Materials:**

Eligible patients had biopsy-proven stage I or II basal cell carcinoma or squamous cell carcinoma (SCC) lesions ≤3 mm deep and ≤8 cm^2^ in area. Patients were administered rhenium-SCT as a resin applied to adhesive foil affixed to the lesion/s, with a dose of 50 Gy to the deepest point. As per the treatment protocol, efficacy was assessed using modified Response Evaluation Criteria in Solid Tumors criteria after 12 months, with planned primary endpoint measuring complete response scheduled for 24 months. Secondary endpoints included patient-reported quality of life (Skin Cancer Index) treatment comfort, cosmesis (visual assessment scale; 1: poor -10: not visible), and toxicity (CTCAE v5.0).

**Results:**

Response rates for 185 treated lesions from 140 patients were 94.1% (174/185) complete response, and 3.2% (6/185) partial response. The remaining lesions were classified as progressive or stable disease in 2.2% (4/185) and 0.5% (1/185), respectively. Quality of life improved by a mean 10.55 (95% CI, 3.79, 17.31) points (100-point scale) from baseline. No patients reported pain or discomfort during treatment. Most patients (88%, 129/147) developed radiation dermatitis as expected, which was predominantly grade 1 or 2 in severity and resolved rapidly. The most common 12-month toxicity in patients was grade 1 hypopigmentation (60.4%; 78/129), while there was no incidence of grade 3 or 4 toxicities at this time. Patient- and clinician-reported cosmesis visual assessment scale outcomes were broadly favorable at 8.1 and 7.7, respectively (10-point scale).

**Conclusions:**

This 12-month interim analysis of EPIC-Skin indicates rhenium-SCT is an effective and well-tolerated treatment for shallow basal cell carcinoma and SCC lesions, yielding favorable safety, cosmesis, and patient-satisfaction outcomes. These outcomes underscore the utility of rhenium-SCT as a single-session, noninvasive treatment for NMSC, offering a safe, effective, and efficient alternative to surgery for patients with functional or cosmetic considerations, and/or comorbidities.

## Introduction

Skin malignancies are the most common cancers reported worldwide.^1^, ^2^, ^3^ Up to 3 million cases of nonmelanoma skin cancer (NMSC), including basal cell carcinoma (BCC) and cutaneous squamous cell carcinoma (SCC), are reported per year worldwide,^3^ although the true incidence is likely much higher. Increasing rates of skin cancer are being driven by ultraviolet light exposure,^4^ with Australia reporting the highest incidence at >1000 per 100,000 individuals.^5^

Occupational exposure to UV is a particular risk, with almost 19,000 deaths attributable to this worldwide in 2019, while rates of work-related NMSC have doubled over the previous 2 decades.^2^ Incidence of NMSC is also higher in men, and increases with age and with immunosuppression.^1^^,^^6^

NMSC lesions are most commonly reported on ultraviolet light-exposed skin, particularly the head and neck regions.^7^ Surgical resection and resultant functional and cosmetic issues in these exposed regions can have a substantial impact on quality of life (QoL).^8^ Prior diagnoses also substantially increase the risk of new lesions, which can lead to significant disease and treatment burden, impacting QoL.^9^

Current treatment options for NMSC include surgery, cryotherapy, immunotherapy, topical and systemic retinoids, chemotherapy, and radiation therapy.^7^^,^^10^^,^^11^

Radiation therapy has demonstrated approximately 90% efficacy in the treatment of NMSC and may be particularly beneficial for patients for whom surgery is contraindicated, who would prefer to avoid surgery, or where surgical excision may result in unsatisfactory cosmetic or functional outcomes.^11^, ^12^, ^13^ However, conventional radiation therapy typically requires multiple daily sessions to balance efficacy with safety.^11^ This is taxing or impractical for many patients and often requires complex planning to achieve optimal outcomes.

There is an opportunity to improve the standard of care for certain shallow NMSC, with respect to efficacy, comfort, and practicality, by offering an innovative radiation therapy treatment that can be delivered quickly in a single outpatient setting. Rhenium-skin cancer therapy (SCT) is an epidermal radionuclide therapy that uses a radioisotope-based [^188^Re] resin to treat shallow (<3 mm deep) NMSC. Rhenium-SCT is currently approved for the treatment of shallow (≤3 mm) BCC and SCC lesions without Peri-neural invasion or high-risk pathology in Australia, New Zealand, South Africa, Europe, and the UK. Approvals in the respective countries were informed by data from several published studies demonstrating efficacy and safety of rhenium-SCT in small cohorts.^14^, ^15^, ^16^, ^17^ To investigate the efficacy, safety, and patient-reported outcomes safety of rhenium-SCT for NMSC, a prospective, multicenter, international, phase 4, single-arm study (EPIC-Skin) was established to collect data in the countries where it is approved and in use for full-fee paying patients, or those with eligible medical plans. We report here the planned 12-month outcomes, which in addition to efficacy, include, toxicity, and patient- and clinician-assessed cosmesis.

## Methods and Materials

The study design, objectives and outcome measures, patient population, and treatment modality have previously been described^18^ (Fig. E1). Briefly, the EPIC-Skin trial (NCT05135052) is an ongoing prospective, multicenter, single-arm, open-label, phase 4 study conducted at 7 sites worldwide, including Australia, South Africa, Germany, Austria, and the United Kingdom. The primary objective of the study as per the treatment protocol is to estimate complete response (CR) rate at 24 month and show noninferiority to historical values for CR rate following surgery and or radiation therapy. Complete response rate for BCC is 91% at 5 years and for SCC 79%. Assuming 1 lesion per subject, a sample size of 120 subjects is sufficient to provide at least 80% power to conclude noninferiority using a one-sided alpha of 0.025 with a margin of 85% for a CR of ≥94%. The primary endpoint is the proportion of lesions achieving a CR at 24 months. Secondary outcome measures are changes from baseline in QoL (adjusted mean change in the Skin Cancer Index (SCI) score from baseline to 6-month follow-up), treatment comfort, and cosmetic outcome.

### Patient population/eligibility

The study was approved by the relevant institutional review boards at each participating site, and all patients provided written informed consent. Eligible patients (>18 years old) were required to have up to 3 biopsy-proven (punch biopsy to validate depth) BCC or well-to-moderately differentiated SCC lesions, with an area ≤8 cm^2^ and a depth ≤3 mm, with clinically node-negative disease. Patients were also required to have a Karnofsky Performance Status ≥70%, provide informed consent, have declined surgery and/or fractionated radiation therapy, or be ineligible for surgery due to tumor location, performance status, or other comorbidities. Patients were excluded from the study if they received prior treatment with surgery, radiation, or laser therapy for their target lesion(s), or if tumors were affecting nerves or bony structures. Patients were additionally excluded if there were clinical concerns of metastatic disease, perineural or lymphovascular invasion, or involvement of the medial canthus, eyelid margin (upper and lower), or vermillion lip. Patients with comorbidities of lupus or scleroderma, basal cell naevus syndrome, xeroderma, vitiligo, albinism, or those receiving ongoing systemic therapy for any malignancy, or in the 4 weeks before study entry were excluded. Patients were also excluded if they were pregnant.

### Treatment

Patients underwent a single treatment by application of [188Re] resin to an adhesive foil (Aerofilm; Aero Healthcare, Australia) affixed to the target lesion (plus a 5 mm margin), which prevents direct contact of the radioactive resin with the patient. The treatment area was determined by the clinician by measuring the lesion using a graphical film. The calculated area, activity applied, and treatment duration were validated by another member of the clinical team. The radioactive resin was directly administered by the appropriately licensed radiation oncologist or nuclear medicine physician local PI. Treatment time required to achieve a 50 Gy dose was determined based on the activity (Mega Becquerel/MBq) of the [188Re]resin applied, tumor depth, and the surface area treated, using VARSKIN 5 calculations with Monte Carlo-based dose point kernels.^19^ Software incorporated into the treatment station cross references the tumor depth, with area and activity applied to determine the requisite treatment time to deliver 50 Gy to the target (deepest) point of the lesion as assessed by punch biopsy. At the cessation of treatment and removal of the film, the patient is screened with a Geiger counter to ensure there is no contamination.

### Assessments

Clinician assessments were unblinded and performed by the treating clinicians for each patient. A modified visual Response Evaluation Criteria in Solid Tumors was used to assess lesion response to treatment,^20^ with diameter measured at baseline, 6-, 12-, and 24-month follow-up. Responses were classified as: CR—complete disappearance of target lesion; partial response (PR)—at least 30% decrease in largest diameter of target lesion; progressive disease (PD)—at least 20% increase in the largest diameter of target lesion; and stable disease—neither sufficient increase or decrease in largest diameter to qualify as PR or PD. Where possible, suspected PR was validated by histological verification.

The SCI^21^ was used to assess QoL. Patient-assessed treatment comfort was recorded on day 14. Cosmetic outcomes were assessed using the Cosmetic Outcome Visual Analogue Scale, by both patient and clinician: this was done using a 0–10 point scale, ranging from 0 = very poor appearance to 10 = no visible wound. Additional analysis was undertaken on the change from baseline to 12 months via a repeated measures model. As subjects could contribute up to 2 values to the analysis (for the 2 follow-up visits), there is an additional level of structure to the data being analyzed. SAS Mixed Procedure was used to account for this additional structure provided by the repeated measurements on subjects over time. The baseline value for each patient was included as a covariate in the model, as was age (under vs over 70 years of age).

Adverse events (AEs) were monitored throughout the study using CTCAE v5.0,^22^ categorized by Medical Dictionary for Regulatory Activities System Organ Class and Preferred Term, by severity and by relationship to rhenium-SCT. AEs of special interest included radiation dermatitis, skin ulceration, alopecia, skin induration, hypo/hyperpigmentation, and telangiectasia.

### Ethics

The study is conducted in compliance with the protocol, the ethical principles originating in or derived from the Declaration of Helsinki and in compliance with IRB/IEC, informed consent regulations, and International Conference on Harmonization Good Clinical Practices Guidelines/ISO 14155:2020.^23^^,^^24^ In addition, all local legal and regulatory requirements will be met.

## Results

### Demographics

Between 2021 and 2024, 187 patients were enrolled into the study and treated with rhenium-SCT. The median age was 71.5 (range: 27-95) years (Table 1). At cut-off (May 30, 2024), enrollment data were available from 184 patients with a total of 254 lesions. The composition of Fitzpatrick skin types among the cohort were: I (41; 23.3%), II (101; 57.4%), III (31; 17.6%), IV (2; 1.1%), V (0; 0%), and VI (1; 0.6%). Most patients had a single lesion (131 patients, 71.2%), while 36 patients had 2 (19.6%), and 17 patients had 3 (9.2%) included in the study. Lesions were primarily located on the head/neck (171/254; 67.3%), with the remaining located on the upper limbs (23/254; 9%), lower limbs (30/254; 11.8%), and torso (30/254; 11.8%). The nose (61/254; 24%), scalp (28/254; 11%), and ear (21/254; 8.3%) lesions made up the predominant head/neck lesions.

**Table 1:** Patient demographics

Characteristic	Overall(N = 189)*	BCC only(N = 149)	SCC only(N = 34)	Both BCC and SCC (N = 4)
Age (y), mean (SD)	70.3 (12.74)	69.7 (12.98)	72.6 (12.00)	74.8 (8.30)
Gender, *n* (%)				
Male	98 (53.3)	74 (50.3)	22 (66.7)	2 (50.0)
Female	86 (46.7)	73 (49.7)	11 (33.3)	2 (50.0)
Ethnicity, *n* (%)				
White	175 (98.9)	141 (99.3)	30 (96.8)	4 (100.0)
Black or African American	2 (1.1)	1 (0.7)	1 (3.2)	0 (0.0)
Fitzpatrick skin type, *n* (%)				
I	41 (23.3)	34 (24.3)	6 (18.8)	1 (25.0)
II	101 (57.4)	80 (57.1)	19 (59.4)	2 (50.0)
III	31 (17.6)	24 (17.1)	6 (18.8)	1 (25.0)
IV	2 (1.1)	2 (1.4)	0 (0.0)	0 (0.0)
V	0 (0.0)	0 (0.0)	0 (0.0)	0 (0.0)
VI	1 (0.6)	0 (0.0)	1 (3.1)	0 (0.0)
Tumor location, *n* (%)				
Head and neck	171 (67.3)			
Upper limb	23 (9.1)			
Lower limb	30 (11.8)			
Torso	30 (11.8)			
Number of lesions, *n* (%)				
One	131 (71.2)			
Two	36 (9.6)			
Three	17 (9.2)			

*Abbreviations*: BCC = basal cell carcinoma; N = number of patients; SCC = squamous cell carcinoma.

⁎ Two patients did not have the type of skin cancer (BCC/SCC) recorded. These patients were both screen failures.

### Response Evaluation Criteria in Solid Tumors assessment at 12 months

For efficacy assessments, there were 140 patients (185 lesions) with a 12-month post-baseline lesion assessment cut-off. The remaining 44 patients (23.9%) were either lost to follow-up or had not yet reached their 12-month time point. Lesion-based response rates were CR for 94.1% (174/185) (95% CI, 89.65%-97.0%) and PR for 3.2% (6/185) of lesions (Table 2). Tumor-type-specific CR was 93.9% (139/148) for BCC and 94.16% for SCC (35/37), indicating comparable outcomes between the subtypes. PD and stable disease were reported for 2.2% (4/185) and 0.5% (1/185) of lesions, respectively.

**Table 2:** RECIST response rates at 12-month follow-up

	All tumors	BCC tumors	SCC tumors
Complete response, *n*/*N* (%)	174/185 (94.1)	139/148 (93.9)	35/37 (94.6)
Partial response, *n*/*N* (%)	6/185 (3.2)	4/148 (2.7)	2/37 (5.4)
Progressive disease, *n*/*N* (%)	4/185 (2.2)	4/148 (2.7)	0/37 (0.0)
Stable disease, *n*/*N* (%)	1/185 (0.5)	1/148 (0.7)	0/37 (0.0)

*Abbreviation*: RECIST = Response Evaluation Criteria in Solid Tumors.

### QoL

There were 128 patients with baseline, 6-month and 12-month SCI QoL assessments for comparison. All SCI subscales and total scores increased from baseline to 12 months by at least 7 points. Increases in scores were similar at 6-month and 12-month follow-up, although 12-month outcomes continued to improve for the total score (8.21 vs 9.23), as well as emotional (10.78 vs 11.39) and social (4.90 vs 7.41) subscales, whereas the improvements in the appearance subscale score was slightly higher at 6-months follow-up (9.44 vs 8.89) (Table 3) (100-point scale).

**Table 3:** Mean positive change from baseline in SCI subscale and total scores at 6-month and 12-month follow-up

	6 Mo				12 Mo			
Number	All tumors (N = 128)	BCC only (N = 99)	SCC only (N = 27)	Both BCC and SCC (N = 2)	All tumors (N = 120)	BCC only (N = 95)	SCC only (N = 23)	Both BCC and SCC (N = 2)
Total score Mean (SD)	8.21 (16.26)	8.88 (17.37)	5.75 (12.11)	7.94 (1.12)	9.23 (17.37)	8.99 (18.31)	10.53 (13.99)	5.56 (2.81)
Emotion subscale Mean (SD)	10.28 (20.16)	10.73 (21.25)	7.91 (16.44)	19.64 (2.53)	11.39 (21.64)	11.23 (22.83)	11.96 (17.48)	12.50 (2.53)
Social subscale Mean (SD)	4.90 (14.59)	5.23 (15.57)	4.07 (11.10)	0.00 (0.00)	7.41 (16.82)	6.62 (17.16)	11.30 (15.76)	0.00 (0.00)
Appearance subscale Mean (SD)	9.44 (23.70)	10.69 (24.60)	5.25 (20.82)	4.17 (5.90)	8.89 (23.29)	9.12 (24.43)	8.33 (19.46)	4.17 (5.90)

*Abbreviations*: BCC = basal cell carcinoma; *N* = number of patients; SCC = squamous cell carcinoma.

**Figure 1:**
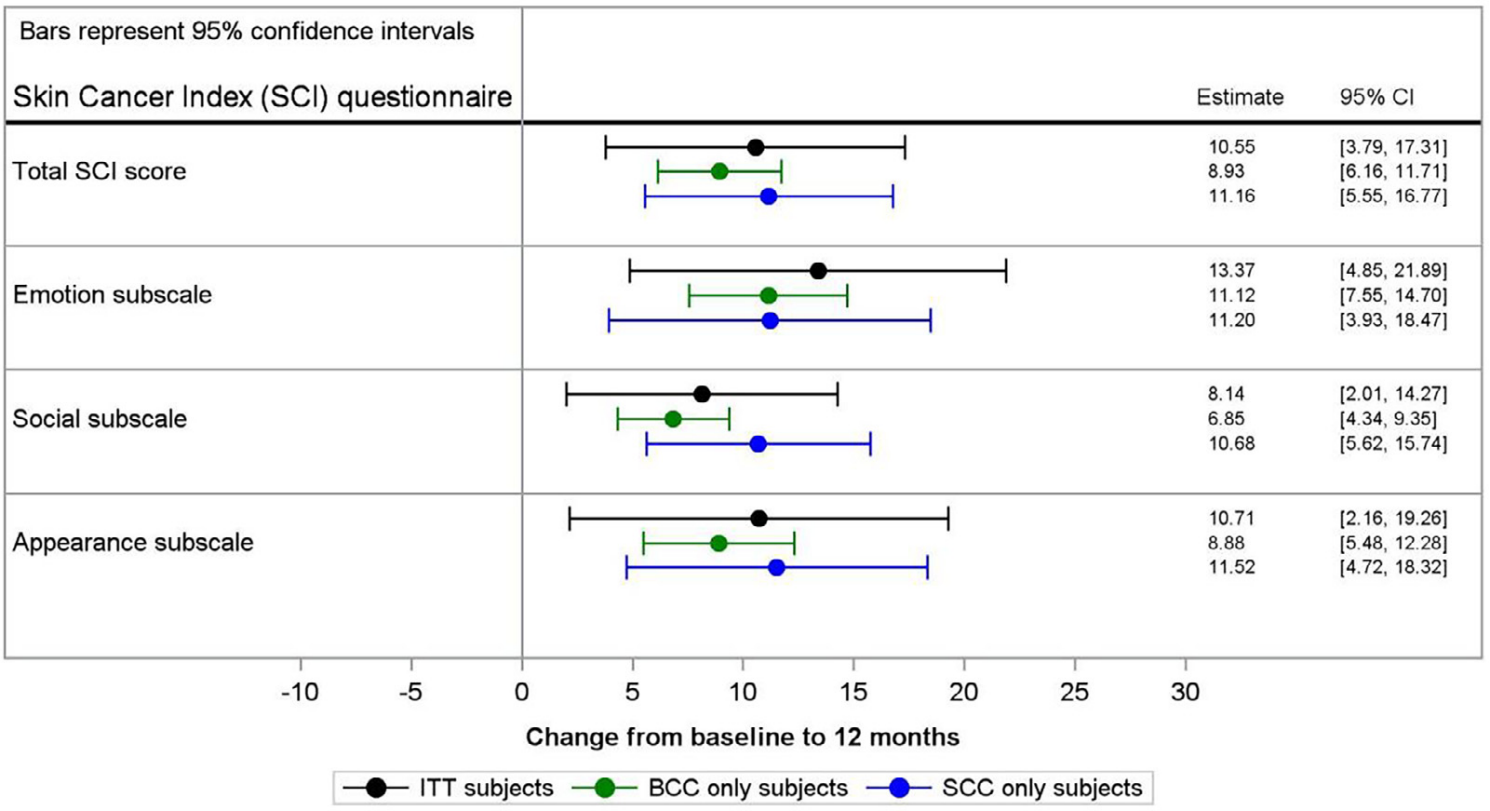
Patient-reported Skin Cancer Index (SCI) quality of life (QoL) assessments. A repeated measures model was used to assess changes in SCI QoL scores at 12-month post-treatment follow-up for “Total” score, as well as “Emotion,” “Social,” and “Appearance” subscales, adjusted to baseline assessments performed before treatment. A positive change indicates an improvement in score from baseline. *Abbreviations*: BCC = basal cell carcinoma; SCC = squamous cell carcinoma.

Significant increases were reported for the overall score and the emotion subscale as assessed via a repeated measures model analysis of ITT subjects. The estimated improvements (95% CI) for the Total, Emotion, Social, and Appearance scores were 10.55 (3.79-17.31); 13.37 (4.85-21.89); 8.14 (2.01-14.27); and 10.71 (2.16-19.26) points, respectively (Fig. 1). Scores were generally higher for BCC than SCC for all subscales. Similar improvements were seen at 6-month follow-up.^2^

### Treatment comfort and Cosmetic assessment

**Figure 2:**
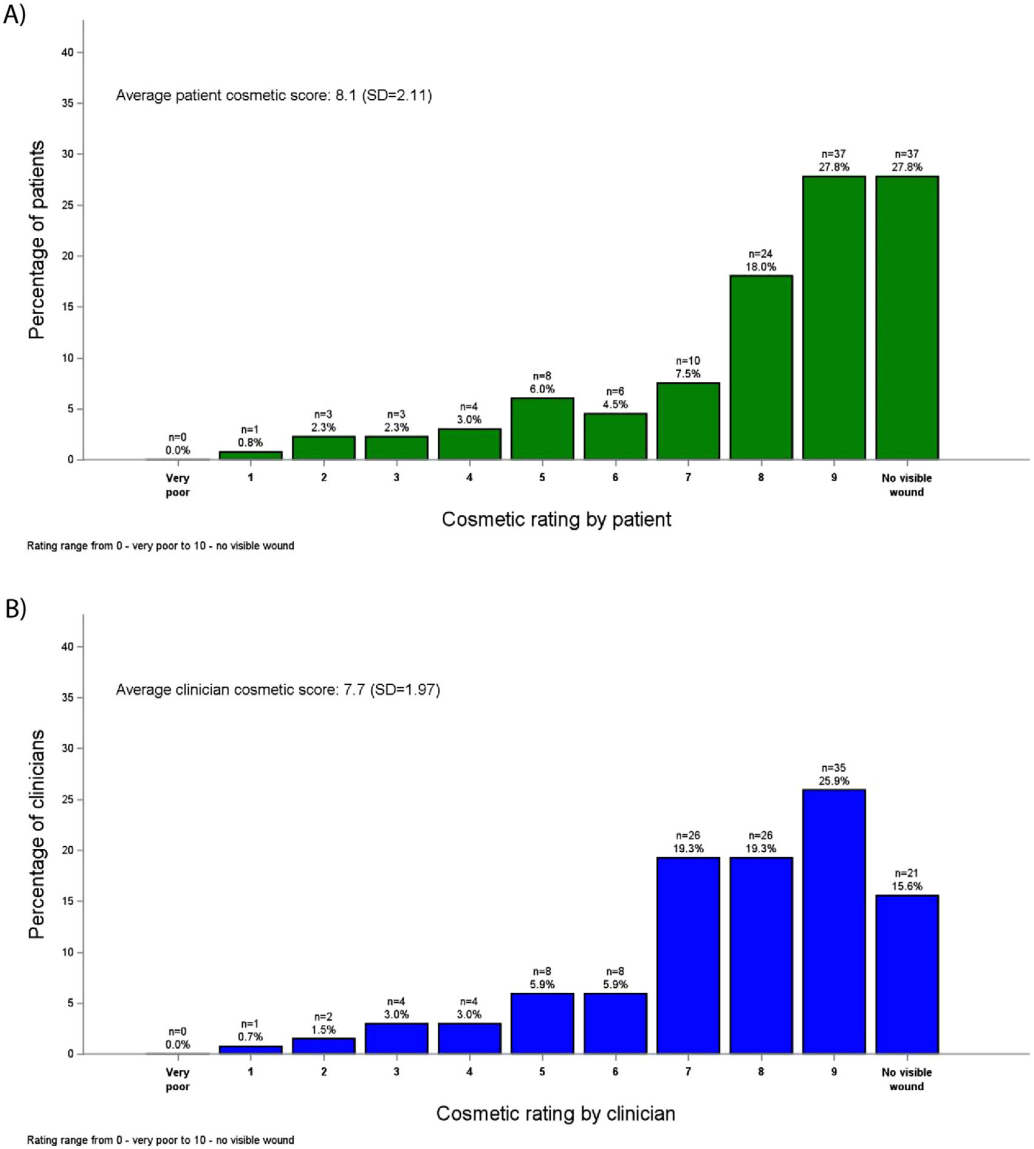
Patient and clinician assessment of cosmetic appearance of wound. Both patients (A) and clinicians (B) provided an assessment of the cosmetic appearance of the wound at 12 months postprocedure. This was performed using a 0–10 point scale, ranging from 0 = very poor appearance to 10 = no visible wound.

All patients who completed the questionnaire reported no pain or discomfort during treatment (no pain: 138/184; 75%; data missing: 46/184, 25%). Missing data are due to patients having to enter certain follow-up measures remotely via the app and missing their time points. Cosmetic Visual Analogue Scale assessments of the treated area were completed and available for 133 patients and 135 clinicians at 12 months, respectively. Patient assessments were slightly higher than clinician assessments, with average scores of 8.1 and 7.7, respectively (Table 4). Over 80% of all scores were 7 or greater (Fig. 2).

**Table 4:** Cosmetic assessment of wound at 12-month follow-up

	Patient assessment			Clinician assessment		
	All tumors (N = 133)	BCC only (N = 108)	SCC only (N = 27)	All tumors (N = 135)	BCC only (N = 110)	SCC only (N = 28)
Cosmetic Score (mean, SD)	8.1 (2.11)	8.1 (1.99)	8.1 (2.55)	7.7 (1.97)	7.6 (1.85)	7.9 (2.43)

*Abbreviations*: BCC = basal cell carcinoma; *N* = number of patients; SCC = squamous cell carcinoma.

Patients with both SCC and BCC are counted in both columns, and once in the all column.

### Safety

There were 142 treatment-related AEs reported in 61/187 (32.6%) of patients during the trial, which were primarily mild skin reactions. There was a total of 6 severe AEs, consisting of dermatitis,^1^ ulceration,^2^ induration,^2^ and radiation skin injury.^1^ There were 3 AEs that lead to study withdrawal; however, none were related to treatment. Additionally, 4 deaths were reported during the study, none of which were related to the treatment.

There were 11 incidences of grade 3 or 4 CTCAE toxicity at any timepoint after treatment. For radiation dermatitis, there were 5 grade 3 and 1 grade 4 incidences reported on day 14, and 1 grade 3 incidence at 6 months. For ulceration, there was one grade 4 incident on day 14, and 2 grade 3 incidents at 6 months. There was one incident of grade 3 induration at 6 months. CTCAE toxicity outcomes were available for 129 patients from 12-month follow-up. The most common reported toxicity was grade 1 hypopigmentation (78/129; 60.5%) while 1 incident (1/129; 0.8%) of grade 2 hypopigmentation was reported. The rates of grades 1 and 2 ulceration, induration, and telangiectasia were 3.1%/2.3%, 11.6%/2.3%, and 10.1%/0%, respectively. There were no reported toxicities greater than grade 2 severity at 12-month follow-up.

## Discussion

Surgical excision of lesions is the mainstay of NMSC treatment; however, complications in up to 5% of patients, comorbidities, and unsatisfactory cosmetic or functional outcomes necessitate other treatment options.^25^ Cryotherapy, immunotherapy, topical and systemic retinoids, chemotherapy, and radiation therapy are all common treatment options for SCC and BCC.^7^^,^^10^^,^^11^ However, there is room for substantial improvement in nonsurgical NMSC treatment outcomes: retinoid therapies have not demonstrated efficacy, tolerability and sustained responses in large-scale clinical trials, despite promising preclinical and case-study reports^10^; chemotherapy regimens carry a higher burden of AEs and are not currently approved for treatment of NMSC, although there is some evidence of efficacy in observational studies^26^; immunosuppression is a risk factor for progression to locally advanced and metastatic disease, and may not be suitable for older patients with comorbidities.^27^

As rhenium-SCT uses a physical barrier between the skin and the radioactive source, it is a noninvasive and painless procedure, enabling treatment of areas with functional or cosmetic considerations. Retrospective studies of rhenium-SCT radiation therapy have shown comparative response rates to surgery, without scarring.^14^, ^15^, ^16^, ^17^, ^28^, ^29^, ^30^ A trial of 43 patients with BCC or SCC reported complete remission for all lesions.^16^ Tietze and colleagues demonstrated 12-month CR rates of 95% from a single session. Patients from this cohort also reported an approximately 2:1 preference for rhenium-SCT over surgical intervention.^17^^,^^31^

Most recently, interim analysis of the EPIC-skin study reported 6-month CR rates >97% from a single session, while demonstrating treatment to be well tolerated.^18^

In the 12-month EPIC-Skin interim analysis herein, we report encouraging efficacy outcomes with a CR rate of 94.1% (95% CI, 89.6%-97.0%), irrespective of tumor type (93.9% for BCC and 94.6% for SCC). The PR rate of 3.2% (6/185) indicates overall treatment responsiveness in >97% of treated lesions. Future histological verification of the 6 lesions with reported PR will be helpful to determine whether these are the original subtype or new disease. This high response rate for rhenium-SCT is consistent with outcomes for fractionated high-dose-rate brachytherapy with median local control rates of 97%,^32^ with the additional benefit of a single treatment session. For the few lesions with a PR, this may render them suitable for salvage surgery that is likely less complicated and/or disfiguring than what would have been necessary initially.

Assessment of QoL, measured by the SCI, demonstrated consistent and continued improvements from baseline, with all SCI subscales and total scores increasing. Slightly higher scores from patient-reported cosmesis over that of clinicians indicate strong patient satisfaction, which is encouraging as many have experience with the cosmetic and functional outcomes from traditional surgical NMSC interventions.

Rhenium-SCT was broadly well tolerated in patients, with most treatment-related AEs occurring at grades 1 and 2 in severity during the acute phase of treatment and resolved rapidly. This reaction profile is in line with the brisk reactions from fractionated radiation therapy. Encouragingly, the longer-term toxicity profile for rhenium-SCT, in terms of type, frequency, and trajectory, is similar to that of conventional radiation therapy that requires fractionation to achieve similar cosmesis and safety outcomes. The total incidence of ulceration and induration out to 12 months were 5.4% and 13.9%, respectively, and align with those from conventional radiation therapy studies at 6.3%^33^ and up to 30%.^34^ These data reinforce the safety and convenience of rhenium-SCT, for which the shallow penetration and sharp dose drop-off profile of [^188^Re]-emitted Beta particles allow for effective and safe treatment in a single session.

Limitations of the analysis include the need for continued follow-up to ascertain long-term recurrence rates, cosmesis, and/or safety profile, which is important for studies of radiation-based treatments. Although consistency between this multicenter global study and previous single-center reports is promising and points to a favorable, durable efficacy and safety profile. Where possible, confirmation of the few reported PRs via histological confirmation of clinically suspicious areas will likely increase the final CR rate. A larger sample size of SCC would be valuable for future studies. For the primary endpoint analysis at 24 months, an increased number of patients reporting for follow-up will be important to make robust efficacy and safety data claims.

## Conclusion

Rhenium-SCT offers a well-tolerated, effective, nonsurgical treatment alternative for certain patients with NMSC with shallow. It yields significant improvements in QoL due to the painless, single-session treatment that controls disease. Recent comparative studies demonstrate that rhenium-SCT also offers patient- and clinician-reported improvements in cosmetic outcomes to surgery, making it an essential consideration for a subset of patients. Additionally, the ability to treat patients with multiple lesions simultaneously underscores the potential utility of rhenium-SCT in managing patients with a high burden of disease. Rhenium-SCT is a safe, and effective treatment for patients with shallow (≤3 mm) NMSC lesions in cosmetically- or functionally sensitive locations, as well as for patients with comorbidities that preclude traditional treatment options.

## Disclosures

This study is a clinical investigation of the OncoBeta product, rhenium-SCT. Siddhartha Baxi, Saima Vohra, and Gerhard Dahlhoff work as medical consultants for OncoBeta. Cody Allison is employed by OncoBeta Therapeutics. Angela Hong and Giuseppe Cardaci have received compensation from OncoBeta for participation in Advisory Boards. MH has received research funding from OncoBeta. The rest of the authors have no conflict of interest to declare. Gerhard Dahlhoff reports administrative support, article publishing charges, equipment, drugs, or supplies, statistical analysis, travel, and writing assistance were provided by OncoBeta GmbH. Giuseppe Cardaci reports article publishing charges, equipment, drugs, or supplies, statistical analysis, and writing assistance were provided by OncoBeta GmbH. Gerhard Dahlhoff reports a relationship with OncoBeta GmbH that includes: board membership, consulting or advisory, and travel reimbursement. Giuseppe Cardaci reports a relationship with OncoBeta GmbH that includes: consulting or advisory, speaking and lecture fees, and travel reimbursement. Cody Allison reports a relationship with OncoBeta Therapeutics Pty Ltd that includes: employment. Saima Vohra reports a relationship with OncoBeta GmbH that includes: consulting or advisory, funding grants, non-financial support, and travel reimbursement. Siddhartha Baxi reports a relationship with OncoBeta GmbH that includes: consulting or advisory, speaking and lecture fees, and travel reimbursement. Martin Heuschkel reports a relationship with OncoBeta GmbH that includes: consulting or advisory and funding grants. Martin Heuschkel reports a relationship with Terumo Deutschland GmbH that includes: consulting or advisory, funding grants, and travel reimbursement. Martin Heusckhel reports a relationship with Novartis that includes: consulting or advisory and funding grants. Martin Heuschkel reports a relationship with Boston Scientific Corporation that includes: consulting or advisory, funding grants, and travel reimbursement. Angela Hong reports a relationship with Telix Pharmaceuticals Limited that includes: consulting or advisory. Siroos Mirzaei reports a relationship with OncoBeta GmbH that includes: travel reimbursement. Nicola Mulholland reports a relationship with OncoBeta GmbH that includes: travel reimbursement.

## Appendix Supplementary materials

SuppE1_EPIC-Skin Trial protocol Image, application 1

EPIC-12-mnth.ARO_SuppFig2 Image, application 2

## Footnotes

Supplementary material associated with this article can be found in the online version at doi:10.1016/j.adro.2025.101802.
