# Supplementary material for: Efficacy, Safety, and Patient Reported Outcomes of Rhenium-Skin Cancer Therapy for Non-Melanoma Skin Cancer: 1-Year Results from the EPIC-Skin Study
Source: Adv Radiat Oncol. 2025 Apr 29;10(7):101802. doi: 10.1016/j.adro.2025.101802 (PMC12197855; doi:10.1016/j.adro.2025.101802)

Supplementary Figure 2: Estimated change from baseline to 6-month follow-ups for SCI subscales, adjusted for baseline subscale scores.

A repeated measures model was used to assess changes in SCI QoL scores at 6-month post-treatment follow-up for “Total” score, as well as “Emotion”, “Social”, and “Appearance” subscales, adjusted to baseline assessments performed prior to treatment.


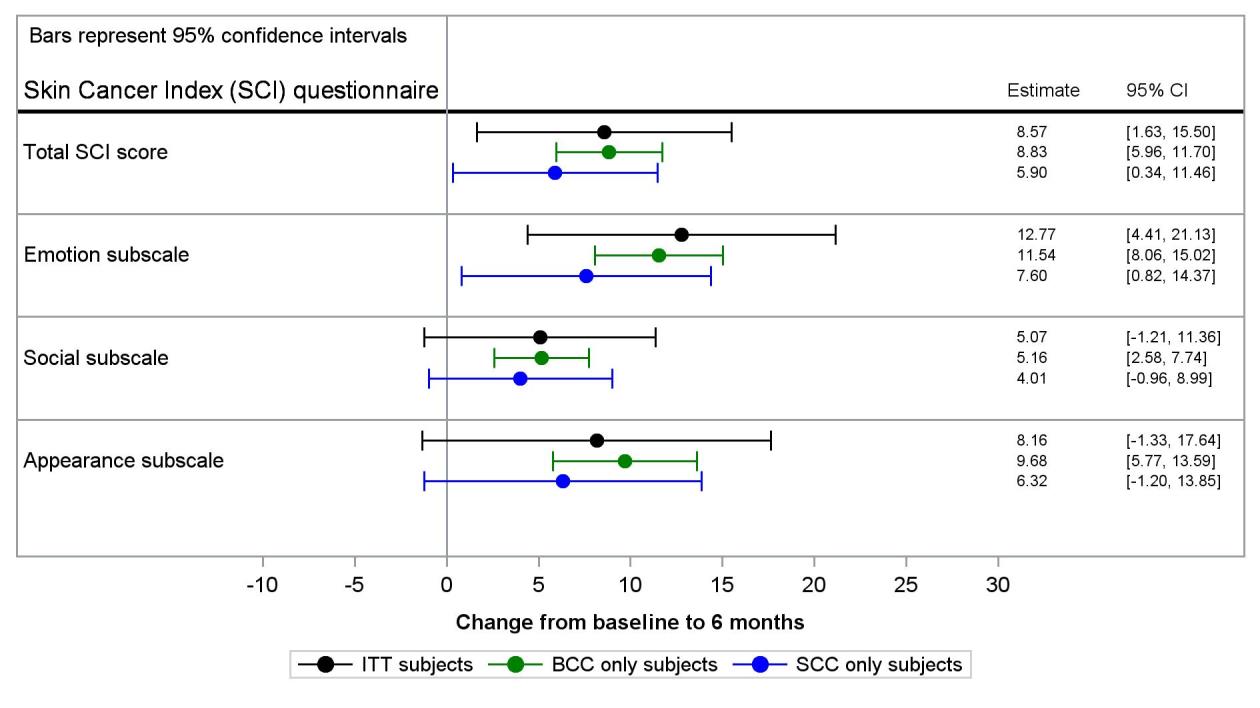

Supplement: SuppE1_EPIC-Skin Trial protocol [file mmc1.docx]
