# Supplementary material for: Efficacy, Safety, and Patient Reported Outcomes of Rhenium-Skin Cancer Therapy for Non-Melanoma Skin Cancer: 1-Year Results from the EPIC-Skin Study
Source: Adv Radiat Oncol. 2025 Apr 29;10(7):101802. doi: 10.1016/j.adro.2025.101802 (PMC12197855; doi:10.1016/j.adro.2025.101802)
Supplement: EPIC-12-mnth.ARO_SuppFig2 [file mmc2.pdf]

DocuSign Envelope ID: 61201B2A-687B-46F1-A8DE-FFB5FF22C98F

CLINICAL STUDY PROTOCOL
EFFICACY OF PERSONALISED IRRADIATION WITH RHENIUM-SKIN CANCER THERAPY (SCT) FOR THE TREATMENT OF NON-MELANOMA SKIN CANCER: A PHASE IV, MULTI-CENTRE, INTERNATIONAL, OPEN LABEL, SINGLE ARM STUDY

**Study Protocol Number:**	OB-RHSCT-101
**Investigational Product:**	Rhenium-Skin Cancer Therapy (SCT)
**Clinical Phase:**	4/ Post Marketing Clinical Follow-up (PMCF)
**Indication:**	Non-melanoma skin cancer
**Sponsor:**	Oncobeta Therapeutics Pty Ltd<br>Level 2, 35-37 Havelock St, West Perth, 6005 Australia
**Sponsor Representative:**	

Version: 2.0
Date 10th November 2021

Previous version:

**Version:**	Version 1.0
**Date:**	29th September 2021

Confidential: The information contained in this document, particularly unpublished data, is in the property or under control of Oncobeta Therapeutics Pty Ltd and is provided to you in confidence as an investigator, potential investigator, or consultant, for review by you, your staff, and an applicable Institutional Review Board or Independent Ethics Committee. The information is only to be used by you in connection with authorized clinical studies of the investigational drug described in the protocol

Protocol Approval Signature Page

This clinical study protocol was subject to critical review and has been approved by the sponsor. The information it contains is consistent with:

- the moral, ethical, and scientific principles governing clinical research as set out in the Declaration of Helsinki and the principles of GCP as described in ICH GCP.

The investigator will be supplied with details of any significant or new findings, including adverse events, relating to treatment with the investigational Device.

Sponsor CEO	DocuSigned by: Nicholas Vetter 248EECD5D56948C...	10/11/2021<br>Date
Sponsor Global Medical Director	DocuSigned by: Gerhard.Dahlhoff 111F9E55BA0E4A2...	10/11/2021<br>Date
Sponsor Country Manager	DocuSigned by: Ken Rikard-Bell 9D638D2C35AB4E9...	11/11/2021<br>Date

Protocol Amendments

Summary of change(s) since last version of protocol

SECTION	DESCRIPTION OF CHANGE
**Title page**	Version number and date
**Appendix 4: Comfort of treatment questionnaire**	Descriptive introduction added: We would like to know how you felt whilst receiving your Rhenium treatment. In particular, any pain that may have been felt. Please note that pressure or discomfort do not constitute pain for the purpose of this question. Also please keep in mind if your pain is due to your lesions, rather than the treatment itself.<br><br>Question 1 clarified by adding wording ‘prior to treatment”
**Appendix 6: Guidance on bleeding post treatment**	Guidance notes added for bleeding, post treatment
**SOA**	**Removal of permissible day rage for day 0: Rhenium-SCT treatment**
**Number of subjects**	Clarification of wording only: 25 Subjects are required per site. Approximately 210 subjects will be recruited (30 subjects per site, from 7 sites), to allow for screening failure or loss to long term follow up.
**Exclusion criteria added**	Participation in another clinical trial or administration of any investigational product or experimental product within 60 days or 5 half-lives (whichever is longer) prior to screening.

DocuSign Envelope ID: 61201B2A-687B-46F1-A8DE-FFB5FF22C98F

Investigator Signature Page

Study Title:

Efficacy of Personalised Irradiation with Rhenium-Skin Cancer Therapy (SCT) for the treatment of non-melanoma skin cancer; a phase IV multi-centre, international, open label, single arm study.

Protocol Version: 2.0 **Date: 10th November 2021**

The undersigned hereby declares his/her consent to performance of the clinical study in compliance with regulations as laid down in this clinical study protocol, in the Declaration of Helsinki, ICH-GCP Guideline, in ISO14155:2020 and applicable national laws and regulations. Changes to this protocol require written agreement of both investigator and sponsor.

The Investigator has acquainted themselves with the results of the studies of the investigational device.

Site Number: ______________________

Principal Investigator: ______________________

Institution: ______________________

Address: ______________________

Investigator:

Printed Name	Signature	Date

Clinical Trial Committee

The study will be overseen by a Clinical trial committee made up of various specialities. The role of the committee is to oversee the study, add expertise where required and comment on any protocol amendments as required. The committee must agree on any adjustments to the protocol and conduct of the study.

Committee members:

Prof. Joe Cardaci	Nuclear Medicine Clinician	Australia
TBC	Radiation Oncologist	Australia
TBC	Dermatologist	Germany
Prof. Dale Bailey	Physicist	Australia

1. SYNOPSIS

Title of Study:

Efficacy of Personalised Irradiation with Rhenium-Skin Cancer Therapy (SCT) for the treatment of non-melanoma skin cancer; a phase IV multi-centre, international, open label, single arm study.

Rationale:

Non-melanoma skin cancer (NMSC), also known as keratinocyte cancer, includes basal cell carcinoma (BCC) squamous cell carcinoma (SCC) and various other less common lesions. NMSC is the most common form of cancer in humans. The most common cause of NMSC is sun exposure, while other predisposing factors include genetic skin conditions and immunosuppressive diseases or treatments. Although a range of definitive treatment options is currently available, these are subject to various limitations of effectiveness, safety, tolerability, and acceptability. New, non-invasive, non-scarring treatment options that can be delivered quickly in an outpatient setting would represent a significant improvement in the standard of care.

Rhenium-SCT is a novel form of brachytherapy for NMSC, which uses the Beta emitter radioisotope Rhenium-SCT. During treatment, the affected area of the skin is covered with a sterile protective foil. Rhenium-SCT is then applied in a matrix on the foil using a special applicator device. The irradiation time required to achieve the desired target dose (50Gy) at the defined penetration depth is calculated based on the radioactivity of the substance being applied and the surface area to be treated. At the conclusion of the calculated irradiation time, the matrix is removed by pulling the foil from the skin. The procedure is highly effective, while also non-invasive, and non-scarring. It can be provided without anaesthesia, over a short period of time and in an outpatient setting. As such, the service offers multiple practical, economic, and subject relevant benefits over existing management options, especially surgical removal of larger lesions. It is expected that treatment with Rhenium-SCT Brachytherapy will have a positive impact on the quality of life of subjects being treated. The reduced scarring and healing time mean subjects can return to their normal lifestyle sooner than would be expected for a surgical treatment.

Rhenium-SCT brachytherapy offers a new modality for treating more serious presentations of NMSC, which is non-invasive, can usually be delivered in a single session, over a short period of time, without the need for anaesthesia, in an outpatient setting. It will be particularly beneficial for lesions which are difficult to treat surgically, due to their size and or location (e.g., ears, nose, eyelids, shins, collarbone).

Clinical trials have shown the procedure to deliver remission rates similar those reported for either conventional or Mohs surgery, without functional mutilation or scarring and associated requirement for corrective or cosmetic repair. The net impact of these benefits for subjects, in terms anaesthesia and infection risk, procedural and post-operative pain, disfiguration and scarring, health related quality of life, healthcare costs, and functional and economic wellbeing are very significant. With this in mind, further clinical trials to demonstrate the ongoing benefit in terms of response rates, will offer efficacy and long-term data, as well as important subject reported outcome measures.

Rhenium-SCT has benefit in patients who are not suitable for surgery, perhaps because of comorbidities or tumour location. In addition, treatment with Rhenium-SCT has benefit as a single treatment and would be favourable for patients for whom several sessions of Radiation Therapy may not be appropriate or acceptable.

Registration Status:

EC Certification – Registered

ISO 13485:2016 – Registered

TGA – Currently undergoing conformity Assessment – In progress

Phase of development:

Phase IV study/ Post Marketing Clinical Follow-up (PMCF)

Classification:

Base Station and Applicator

The Rhenium-SCT application system, constituted by the Base Station and the Applicator, can be classified as a class I device as per the Medical Device Regulation (MDR) Annex VIII Rule 1.

Base Station REF: 0200

Applicator REF: 0100

Measurement Station

According to the classification rules set out in EU MDR (EU 2017/745), MST is a Class IIb device.

Measurement Station REF: 0401 or 0404

Carpoules filled with radioactive compound (Re-188 compound)

According to the Medical Device Regulation Annex VIII classification rule 9, paragraph 3, the OncoBeta Rhenium-188 compound can be classified as a medical device class IIb.

Radioactive carpoules REF: 0300

Please note that we do not specify the model and/or type directly, but this is linked with the reference number and the serial numbers that we give to the different stations/devices.

Protective Foil

The foil is a 3rd party product and it is a medical device classified as class Is.

Model / Type:

Oncobeta Rhenium-SCT

Subject Population:

Subjects with stage I or II NMSC (histologically confirmed BCC or SCC [SCC Well to Moderately differentiated])

Studied duration (planned):

12 months, with 24 months long-term follow-up

Number of subjects (planned):

Number of sites:

7 sites in total: Australia (4 sites) and Europe: (Germany, Austria and UK 1 site each)

25 Subjects are required per site. Approximately 210 subjects will be recruited (30 subjects per site, from 7 sites), to allow for screening failure or loss to long term follow up.

Type of study, study design:

Prospective, multicentre, single arm, open-label, phase IV study/ Post Marketing Clinical Follow-up (PMCF) study.

An interim analysis is planned once 50% of overall (total) subjects have been treated and have had a 6 month follow up visit.

Study objectives:

Primary objective:

- To assess the proportion of lesions achieving complete response (CR) as per MODIFIED VISUAL RECIST criteria

Secondary objectives:

- To assess change from baseline in Quality of Life (QoL)
- To assess treatment comfort
- To assess cosmetic outcomes

Primary endpoint:

- The number and percentage of lesions achieving CR during the study

Secondary endpoints:

- Adjusted mean change from baseline in QoL score at each timepoint
- Frequency and percentage of subjects reporting each option on the treatment comfort questionnaire
- Adjusted mean cosmetic outcome score.

Safety endpoint:

- Adverse events by severity and by relationship to Rhenium-SCT, including radiation dermatitis, dry skin, skin ulceration, alopecia, skin induration, hypo/hyperpigmentation, and telangiectasia.

Criteria for inclusion:

1. Stage I or II BCC or SCC (SCC Well to Moderately Differentiated), and clinically node negative disease
2. Confirmed Histology, and with depth of lesion noted
3. Subjects with up to 3 lesions suitable to enter the study (subjects with more than 3 lesions are not excluded from the study, however 3 target lesions are determined for study evaluation only.)
4. Subjects able and willing to comply with the requirements of the study
5. Age >=18 years
6. Informed Consent signed by the subject consenting to undergo the study
7. Lesions up to $8cm^2$
8. Lesions with a depth up to 3mm confirmed on biopsy report AND deemed appropriate clinically by treating clinician
9. Subjects who are not deemed suitable for surgery, for example due to tumour location, performance status or other comorbidities as deemed relevant by the treating clinician
10. Patients who may have declined Surgery and/or fractionated Radiation Therapy

Criteria for exclusion:

1. Inability to personally provide written informed consent or to understand and collaborate throughout the study
2. Inability or unwillingness to comply with study requirements
3. Prior treatment with surgery or radiation therapy for their target lesion(s)
4. Depth of lesion greater than 3mm as defined by Biopsy and/or clinical assessment
5. Lupus and Scleroderma
6. Basal cell naevus syndrome, xeroderma, vitiligo and albinism
7. Prior laser at the tumour site
8. Malignant melanoma systemic therapy ongoing
9. Any ongoing treatment for malignancy, or in the last 4 weeks prior to study entry
10. A tumour affecting nerves or bony structures
11. Clinical concern of metastatic disease
12. Pregnancy and/or Lactation
13. Pathological exclusions: Perineural Invasion, Lymphovascular invasion
14. Anatomical exclusions: NMSC's of the Medial canthus, eyelid margin (upper and lower), Vermillion lip
15. Participation in another clinical trial or administration of any investigational product or experimental product within 60 days prior to screening.

Investigational Device:

Rhenium-SCT

Treatments, dose, mode of administration, duration of treatment:

Rhenium-SCT (50Gy dose at target depth) will be administered on Day 0, as a single treatment, by application on a protective foil applied over the area to be treated, such that the radioactive material does not come into contact with the subject. The depth of lesion is determined before treatment by Histology (biopsy).

The lesion is marked out prior to treatment with a 5mm safety margin. The irradiation time required to achieve the desired target dose at the defined penetration depth is calculated based on the amount of radioactivity of the substance being applied, the surface area to be treated and the depth of the lesion. The dose at target depth, the deepest point of the lesion, is defined as 50 Gy. This dose, also known as target dose, ensures the mortality of all tumorous cells at the target depth. After the calculated irradiation time has elapsed, the Rhenium-SCT is removed by pulling the foil from the skin. A full user guide will be provided to the site.

Criteria for evaluation:

Efficacy:

1. Rate of complete response (CR) for subjects treated with Rhenium-SCT at 12 months after treatment

   MODIFIED VISUAL RECIST (adapted from Response Evaluation Criteria in Solid Tumours) CRITERIA 1.1

   - Complete response (CR): disappearance of the target lesion/s
   - Partial Response (PR): ≥ 30% reduction of the larger initial diameter of the target lesion or sum of the larger initial diameters of the target lesions
   - Progressive Disease (PD): ≥ 20% increase in the largest initial diameter of the lesion or sum of the larger initial diameters of the target lesions
   - Stable Disease (SD): Neither partial response or progressive disease
2. Quality of Life (Baseline: prior to therapy, 6 months after therapy, and 12 months after therapy) – NMSC QOL questionnaire
3. Overall response rate (ORR)
4. Comfort of treatment with Rhenium-SCT using short questionnaire
5. Cosmetic outcome (measured 12 months after therapy) using VAS. Both subject and clinician assessed.

Safety

Common Terminology Criteria for Adverse Events (CTCAE) 5.0 grading for acute and late effects, relevant to treatment:

- Radiation dermatitis
- Skin ulceration
- Alopecia
- Skin induration
- Hypo/hyperpigmentation

- Telangiectasia

Statistical methods:

Sample size calculation:

The primary objective of the study is to estimate CR rate and show non-inferiority to historical values for CR rate following surgery and or radiotherapy. CR rate for BCC is 91% at 5 years[1] and for SCC 79%[2]. Review of the published papers for brachytherapy shows the split between BCC and SCC is 2:1[3], 1.68:1[4] and 2.1:1[5].

Based on previous studies, the CR rate for BCC and SCC is expected to be close to 100% following Rhenium-SCT treatment. For simplicity, assuming 1 lesion per subject, a sample size of 120 subjects is sufficient to provide at least 80% power to conclude non-inferiority using a one-sided alpha of 0.025 under the following circumstances (Table 1).

In this study, approximately 210 subjects will be recruited to allow for screening failure or loss to long-term follow-up) and ensure a minimum of 120 patients are included in the analysis.

Table 1. Sample size calculation

Non-inferiority margin	Assumed CR rate
85%	≥94%
86%	≥94%
87%	≥95%
88%	≥96%
89%	≥97%
90%	≥97%

CR: Complete response

Analysis Populations

Intent-to-Treat (ITT) Population

The ITT Population is defined as all subjects who were enrolled into the study, received treatment and have at least one post baseline value for efficacy data.

Safety Population

The Safety Population is defined as all subjects in the ITT and for whom there is at least one value for safety data.

Efficacy analysis:

Primary efficacy endpoint: Complete Response (CR).

The number of lesions treated per subject will be summarised.

The number (and percentage) of lesions in each category (CR, PR, SD and progressive PD) at each timepoint (6 months, 12 months and 24 months) and the overall best response will be presented. The percentage of lesions achieving CR during the study (i.e., CR being the best overall response) will be estimated and presented with exact 95% confidence limits overall and by tumour type.

Secondary efficacy endpoints: Change from baseline in QoL, comfort of treatment, and cosmetic outcomes.

The QoL questionnaire will be scored according to the author's instructions. Change from baseline in score will be calculated. Descriptive statistics will be presented for actual score and change from baseline by timepoint, and overall and by tumour type (BCC/SCC). A mixed model will be fitted with change from baseline as the outcome variable. Baseline score will be included as a covariate and timepoint (6 months/12 months) tumour type, tumour stage as factors. Other relevant prognostic factors assessed at baseline will be included. From the model the adjusted mean change at each timepoint will be obtained and presented with 95% confidence limits. Adjusted mean change will be estimated overall and by tumour type.

Comfort with treatment is being assessed using a questionnaire. The frequency (and percentage) of subjects reporting each option for ach question will be presented.

Cosmetic outcome (subject and clinician rated) is being assessed at 12 months and 24 months using a visual analogue score (VAS). Descriptive statistics will be presented overall and by tumour type (BCC or SCC). A mixed model will be fitted with VAS as the outcome variable. Tumour type and tumour stage will be included as factors. Other relevant prognostic factors assessed at baseline will be included. From the model the adjusted mean score will be obtained and presented with 95% confidence limits. Adjusted mean score will be estimated overall and by tumour type.

TABLE OF CONTENTS

Protocol Approval Signature Page 2
Protocol Amendments 3
Summary of change(s) since last version of protocol 3
1. SYNOPSIS 6
TABLE OF CONTENTS 13
List of Abbreviations and Definition of Terms 18
2. ETHICAL CONSIDERATIONS AND ADMINISTRATIVE PROCEDURES 19
2.1 Regulatory Authority Approval 19
2.2 Investigator Responsibilities 19
2.2.1 Good Clinical Practice 19
2.2.2 Ethical Conduct of the Study and Ethics Approval 19
2.2.3 Informed Consent 19
3. INVESTIGATORS AND STUDY ADMINISTRATIVE STRUCTURE 21
4. INTRODUCTION 22
4.1 Background 22
4.2 Investigational Device 22
4.2.1 Rhenium-SCT 22
4.3 Clinical Data 23
4.4 Study Design Rationale 23
5. STUDY OBJECTIVES AND ENDPOINTS 25
5.1 Primary Objective 25
5.2 Primary Endpoint 25
5.3 Secondary Objectives 25
5.4 Secondary Endpoints 25
5.5 Safety Endpoint 25
6. STUDY DESIGN 26
6.1 Overall Study Design 26
7. SELECTION OF STUDY POPULATION 27
7.1 Inclusion Criteria 27
7.2 Exclusion Criteria 28
7.3 Withdrawal and Discontinuation 28

DocuSign Envelope ID: 61201B2A-687B-46F1-A8DE-FFB5FF22C98F

7.3.1 Subject Withdrawal 28
7.3.2 Termination of the Study 29
8. STUDY TREATMENTS 30
8.1 Study Treatment 30
8.1.1 Treatments Administered 30
8.1.2 Identity of Investigational Products 30
8.1.3 Packaging and Labelling 31
8.1.4 Handling and Storage 32
8.1.5 Compliance and Accountability 33
8.1.6 Prior and Concomitant Therapy 34
8.1.7 Unacceptable/Prohibited Therapy 34
8.1.8 Blinding 34
9. STUDY PROCEDURES AND ASSESSMENTS 35
9.1 Study Flow and Visit Schedule 35
9.1.1 Informed Consent Visit / Screening and baseline (Day -30 to Day -1) 38
9.1.2 Treatment Period 38
9.1.2.1 Day 0 38
9.1.2.2 Day 14 38
9.1.2.3 Day 30 Phone call 39
9.1.3 Follow-up 39
9.1.4 Premature Study Termination 40
9.2 Efficacy Assessments 41
9.2.1 Response Assessments 41
9.2.2 Trial App 41
9.2.3 Subject Reported Outcome Measures (PROMs) 42
9.3 Demographics and Medical History 42
9.4 Safety Assessments 43
9.4.1 Clinical Laboratory Evaluation 43
9.4.2 Pregnancy Test 43
9.4.3 Pharmacokinetics 43
9.4.4 Pharmacodynamics 43
9.4.5 Pharmacogenetics 43

10. PHARMACOVIGILANCE 44
10.1 Definition of Adverse Events 44
10.2 Definition of Serious Adverse Events (SAEs) 44
10.3 Definition for Medical Device 45
10.3.1 Adverse Device Effect (ADE) 45
10.3.2 Device Deficiency (DD) 45
10.3.3 Incident and Serious Incident 45
10.3.4 Unanticipated Serious Adverse Device Effect (USADE) 45
10.4 Period of Observation 46
10.5 Documentation and Reporting of Adverse Events 46
10.5.1 Nature 47
10.5.2 Severity 47
10.5.3 Duration 47
10.5.4 Relationship 47
10.5.5 Outcome 48
10.6 Documentation and Reporting of Serious Adverse Events 49
10.7 Reporting of Pregnancy and Lactation to the Sponsor 50
10.8 Deaths 50
10.9 Expedited Reporting of Adverse Events 50
10.9.1 Development Safety Update Reports 50
10.9.2 Medical Device Vigilance Reporting 50
11. STATISTICAL METHODS 52
11.1 Determination of Sample Size 52
11.2 Statistical and Analytical Plans 52
11.3 Summary of Variables 52
11.4 Data Sets to be Analysed 53
11.4.1 Safety Analysis Set 53
11.4.2 Full Analysis Set 53
11.4.3 Per-Protocol Set 53
11.5 Summary of Subject Disposition 53
11.6 Baseline and Background Characteristics 53
11.7 Efficacy Analyses 53
11.8 Safety Analyses 54

11.8.1.1 Adverse Events 54
11.8.2 Clinical Laboratory 54
11.8.3 Physical Examination and Other Safety Measures 54
11.9 Subgroup Analyses 54
11.10 Interim Analyses 55
11.11 Handling of Dropouts and Missing Data 55
12. DATA COLLECTION, HANDLING, AND RECORD KEEPING 56
12.1 Generation of Data Base 56
12.2 Data Collection 56
12.2.1 Source Data 57
12.3 Data Management 57
12.4 Data Protection 57
13. QUALITY ASSURANCE 60
13.1 Data Monitoring 60
13.2 Audits and Inspections 61
14. ETHICAL AND LEGAL REQUIREMENTS 62
14.1 Independent Ethics Committee 62
14.2 Ethical Conduct of the Study 62
14.3 Changes in the Conduct of the Study 62
14.4 Subject Information and Consent 62
14.5 Confidentiality 63
14.6 Finance, Subject Insurance Coverage and Investigator Indemnity 63
14.7 Publication of Study Results 64
REFERENCE LIST 65
APPENDICES 67
Appendix 1: CTCAE grading Version 5.0 67
Appendix 2: QOL Questionnaire 68
Appendix 3: VAS Cosmetic outcome (clinician and subject rated) 69
Appendix 4: Comfort of treatment questionnaire 70
Appendix 5: Modified Visual RECIST 71
Appendix 6. Guidance notes on Bleeding post treatment 73

List of In-Text Tables

Table 1. Sample size calculation 11
Table 2. Study Assessments and Procedures Schedule 36
Table 3. Sample size calculation 52
Table 4. Evaluation of best overall response 72
Table 5. Example Modified Visual RECIST data collection: 73

List of In-Text Figures

Figure 1. Rhenium-SCT Carpoule 333

List of Abbreviations and Definition of Terms

AE	Adverse Event
BCC	Basal Cell Carcinoma
CR	Complete Response
CE	Conformitè Europëenne
CRA	Clinical Research Associate
CRO	Contract Research Organisation
CTCAE	Common Terminology Criteria for Adverse Events
DSUR	Development<br>Update Safety Reports
eCRF	Electronic Case Report Form
EMA	European Medical Association
FAS	Full analysis set
GCP	Good Clinical Practice
GDPR	General Data Protection Regulation
ICH	International Conference on Harmonization
IEC	Institutional Ethics Board
IMP	Investigational Medicinal Product
IND	Investigational New Drug Application
IRB	Institutional Review Board
ITT	Intent-to-treat
ISO 14155:2020	Standard for Clinical investigation of medical devices for human subjects — Good clinical practice
NMSC	Non-Melanoma Skin Cancer
PD	Progressive Disease
PR	Partial Response
PT	Preferred term
QoL	Quality of Life
REC	Radioactive Rhenium-SCT compound
RECIST	Response Evaluation Criteria in Solid Tumours
RSI	Reference safety information
SAE	Serious Adverse Event
SCC	Squamous Cell carcinoma
SCT	Skin cancer therapy
SD	Stable Disease
SOC	System organ class
SOP	Standard Operating Procedure
SUSAR	Suspected Unexpected Serious Adverse Reaction
TGA	Therapeutic Goods Administration Australia
UK	United Kingdom
US	United States
VAS	Visual Analogue Scale
WHO-UMC	World Health Organisation-Uppsala Monitoring Centre

2. ETHICAL CONSIDERATIONS AND ADMINISTRATIVE PROCEDURES

2.1 Regulatory Authority Approval

The sponsor will obtain approval to conduct the study from the appropriate regulatory agency in accordance with any applicable country-specific regulatory requirements before the study is initiated at a study centre in that country.

2.2 Investigator Responsibilities

2.2.1 Good Clinical Practice

The Investigator will ensure that this study is conducted in full conformance with the principles of the "Declaration of Helsinki" (as amended in Tokyo, Venice, Hong Kong, South Africa, and Edinburgh) or with the laws and regulations of the country in which the research is conducted, whichever affords the greater protection to the individual. The study must fully adhere to the principles outlined in "Guideline for Good Clinical Practice" International Council on Harmonisation (ICH) Tripartite Guideline or with local law if it affords greater protection to the subject. For studies conducted in the United States of America (US) or under US Investigational New Drug Application (IND), the Investigator will additionally ensure that the basic principles of "Good Clinical Practice" as outlined in the current version of 21 Code of Federal Regulations (CFR), subchapter D, part 312, "Responsibilities of Sponsors and Investigators", part 50, "Protection of Human Subjects", and part 56, "Institutional Review Boards", are adhered to.

In other countries where "Guideline for Good Clinical Practice" exists the Sponsor and the Investigators will strictly ensure adherence to the stated provisions.

2.2.2 Ethical Conduct of the Study and Ethics Approval

This Protocol and any accompanying material provided to the subject (such as subject information sheets or descriptions of the study used to obtain informed consent) as well as any advertising or compensation given to the subject, will be submitted by the Investigator to an Institutional Review Board (IRB)/Institutional Ethics Committee (IEC). Approval from the Committee must be obtained before starting the study and should be documented in a letter to the Investigator specifying the date on which the committee met and granted the approval.

Any modifications made to the Protocol after receipt of the IEC approval must also be submitted by the Investigator to the Committee in accordance with local procedures and regulatory requirements.

When no local review board exists, the Investigator is expected to submit the Protocol to a regional committee. If no regional committee exists, the Sponsor will assist the Investigator in submitting the Protocol to an appropriate Ethics Review Committee.

It is the understanding of the Sponsor that this Protocol (and any modifications) as well as appropriate consent procedures will be reviewed and approved by an IRB. This board must operate in accordance with the current Federal Regulations. A letter or certificate of approval will be sent by the Investigator to the Sponsor before initiation of the study, and also whenever subsequent modifications to the Protocol are made.

2.2.3 Informed Consent

It is the responsibility of the Investigator, or a person designated by the Investigator (if acceptable by local regulations), to obtain written informed consent from each subject participating in this study, after adequate explanation of the aims, methods, anticipated benefits, and potential hazards of the study. For subjects not qualified or incapable of giving

DocuSign Envelope ID: 61201B2A-687B-46F1-A8DE-FFB5FF22C98F

legal consent, written consent must be obtained from the legally acceptable representative. In the case where both the subject and his/her legally acceptable representative are unable to read, or unable to understand due to language barriers, an impartial witness should be present during the entire informed consent discussion. After the subject and representative have orally consented to participation in the study, the witness' signature on the form will attest that the information in the consent form was accurately explained and understood. The Investigator or designee must also explain that the subjects are completely free to refuse to enter the study or to withdraw from it at any time, for any reason. The Case Report Forms (CRFs) for this study contain a section for documenting informed subject consent, and this must be completed appropriately. If new safety information results in significant changes in the risk/benefit assessment, the consent form should be reviewed and updated if necessary. No study related activities should be conducted on a subject until after obtaining informed consent.

3. INVESTIGATORS AND STUDY ADMINISTRATIVE STRUCTURE

This study will be conducted at sites in Australia, Europe, and UK.

The name, telephone and email addresses of the Medical Monitor and other contact personnel at the Sponsor are listed in the regulatory binder provided to each site.

4. INTRODUCTION

4.1 Background

Non-melanoma skin cancer (NMSC), also known as keratinocyte cancer, includes basal cell carcinoma (BCC) squamous cell carcinoma (SCC) and various other less common lesions[6]. NMSC is the most common form of cancer, with the highest incidence seen in Australia (incidence >1000/100,000 person years for BCC)[7], with almost one third of all cancer cases attributable to skin cancer . Cases are increasing globally, with the greatest increase seen in the UK (incidence 76.21/100,000 person-years for BCC and 22.65/100,000 person-years for SCC)[7]. Although mortality is generally low compared to other common carcinomas, NMSC results in a considerable impact on quality of life (QoL) particularly since most lesions develop on commonly exposed areas of skin, such the face and arms[7]. Additionally, NMSC represents an increasing economic burden, with costs rising annually from a predicted £180 million in the UK alone[8].

The most common cause of NMSC is sun exposure, while other predisposing factors include age, genetic skin conditions, and immunosuppressive diseases or treatments, with higher rates seen in men[9]. Although a range of definitive treatment options is currently available, these are subject to various limitations of effectiveness, safety, tolerability, and acceptability. New, non-invasive, non-scarring treatment options that can be delivered quickly in an outpatient setting would represent a significant improvement in the standard of care.

4.2 Investigational Device

4.2.1 Rhenium-SCT

Rhenium-SCT is a novel form of brachytherapy for NMSC, which uses the Beta emitter radioisotope Rhenium-SCT. Rhenium-SCT is obtained from a 188W/188Re generator and has a half-life of about 17 hours, which means that it decays continuously over a short period of time. Rhenium-SCT particles produced from the elution of the 188W/188Re generator are bound to a fluid matrix resulting in the compound inside REC (radioactive Rhenium-SCT compound) carpoules. The Rhenium-SCT isotope used for the Rhenium-SCT treatment is a β-emitter with main decay energies of maximum 2120 keV with 71% intensity and 1965 keV with 25% intensity (765 keV on average). In addition to its main β-emissions, Rhenium-SCT emits γ-radiation of 155 keV with a 15% intensity, as well as other β- and γ-emissions of different energies.

During treatment, the affected area of the skin is covered with sterile protective foil. Rhenium-SCT is then applied in a matrix on the foil using a special applicator device. The irradiation time required to achieve the desired target dose (50Gy) at the defined penetration depth is calculated based on the radioactivity of the substance being applied and the surface area to be treated. After the calculated irradiation time, the matrix is removed by pulling the foil from the skin. The procedure is highly effective, while also non-invasive, and non-scarring. It can be provided without anaesthesia, over a short period of time in an outpatient setting. As such, the service offers multiple practical, economic, and subject relevant benefits over existing management options, especially surgical removal of larger lesions. It is significant that treatment with Rhenium-SCT Brachytherapy will have a positive impact on the quality of life

of subjects being treated. The reduced scarring and healing time mean subjects can return to their normal lifestyle sooner than would be expected for a surgical treatment.

4.3 Clinical Data

Clinical studies conducted so far have shown the procedure to deliver response rates similar those reported for either conventional or Mohs surgery, without functional mutilation or scarring and associated requirement for corrective or cosmetic repair[4,10,11]. Histologically confirmed responses have been reported in patients with keloids, benign dermal tumours, and non-melanoma skin cancers[13,14]

Rhenium-188 brachytherapy has been successfully used in a variety of clinical trials over the past 15 years. An early trial of 53 patients with histologically confirmed skin carcinomas (BCC or SCC) received treatment with Rhenium-188. A complete response was obtained following a single application in 82% of the cases, with the remaining patients reporting a complete response after additional applications. No clinical relapses were observed (mean follow-up 51 months)[5].

In a more recent trial of 52 patients with NMSC (treating a total of 55 lesions, including 32 BCC and 19 SCC), complete remission was reported for all lesions. Importantly, no side effects were reported. Additionally, no recurrence occurred during the follow-up period (mean 288 days)[3].

The non-invasive methodology of Rhenium-188 treatment is particularly important when surgery is not possible or desirable (for example tumours on the face or mucosal tissues). In a study treating 15 patients with SCC of the penis, 12 patients reported a complete response. Additionally, the treatment was painless and the anatomical integrity of the organ was maintained[15].

Brachytherapy with Rhenium-188 has also been investigated in patients suffering from extramammary Paget's disease (EMPD). Five patients received either 1 or 2 treatments, with all patients reporting a complete response; however, 4 patients later reported a relapse[10].

The net impact of these benefits for subjects, in terms anaesthesia and infection risk, procedural and post-operative pain, disfiguration and scarring, health related quality of life, healthcare costs, and functional and economic wellbeing are substantial[12]. Further clinical trials to demonstrate the ongoing benefit in terms of response rates will offer efficacy and long-term data, as well as important subject reported outcome measures.

4.4 Study Design Rationale

Rhenium-SCT brachytherapy offers a new modality for treating presentations of NMSC, which is non-invasive, can usually be delivered in a single session, over a short period of time, without the need for aesthesia, in an outpatient setting. It will be particularly beneficial for lesions which are difficult to treat surgically, due to their size and or location. In order to demonstrate efficacy of Rhenium-SCT as well as the important patient reported outcomes, this study design has been built with extensive input from treating clinicians, as well as experienced sites using Rhenium-SCT. Efficacy will be measured using the robust endpoint

DocuSign Envelope ID: 61201B2A-687B-46F1-A8DE-FFB5FF22C98F

of Response Rate. Patient reported outcome measures will include those that are important to patients with NMSC's. These include QoL, Cosmetic outcome and comfort of treatment.

5. STUDY OBJECTIVES AND ENDPOINTS

5.1 Primary Objective

- To assess the proportion of lesions achieving CR

5.2 Primary Endpoint

- To assess the number and percentage of lesions achieving CR during the study

5.3 Secondary Objectives

- To assess change from baseline in Quality of Life (QoL)
- To assess treatment comfort
- To assess cosmetic outcome

5.4 Secondary Endpoints

- Adjusted mean change from baseline in QoL score at each timepoint
- Frequency and percentage of subjects reporting each option on the treatment comfort questionnaire
- Adjusted mean Cosmetic Outcome Score will be estimated overall and by tumour type

5.5 Safety Endpoint

- Safety: Adverse events (AEs) by severity and by relationship to Rhenium-SCT, including radiation dermatitis, skin ulceration, alopecia, skin induration, hypo/hyperpigmentation, and telangiectasia.

6. STUDY DESIGN

6.1 Overall Study Design

This is a prospective, multicentre, single arm, open-label, phase IV/ Post Marketing Clinical Follow-up (PMCF), study consisting of:

- A screening period of up to 30 days duration, to ensure that the subject is eligible for the study
- A follow-up period of 24 months. Each subject will remain in the study for 24 months from the time of their treatment with Rhenium-SCT. Subjects requiring further or alternative treatments for the target lesions during the trial period, will leave the study.

The study is completed with the last visit of last subject.

Details on all study visits can be found in the time and events schedule in Section 9.

An interim analysis is planned once 50% of subjects have recorded a 6-month follow-up visit.

7. SELECTION OF STUDY POPULATION

The study population will consist of male and female subjects aged 18 years or older with stage I or II non-melanoma skin cancer (histologically confirmed BCC or SCC).

Non-melanoma skin cancer is common in men and women, with peak age varying by geographical region: In the UK almost half of cases are diagnosed in those aged 75 and over; In Australia almost two thirds are aged under 70. Therefore, it is planned to enrol adult subjects without restrictions in terms of gender and age. The lack of an upper age limit is justifiable since particularly vulnerable, frail subjects are already excluded from study participation by eligibility criteria.

Rhenium-SCT has benefit in patients who are not suitable for surgery, perhaps because of comorbidities or tumour location. In addition, treatment with Rhenium-SCT has benefit as a single treatment and would be favourable for patients for whom several sessions of Radiation Therapy may not be appropriate or acceptable.

The investigator must ensure that all subjects being considered for the study meet the following inclusion and exclusion criteria. No additional exclusions should be applied by the investigator. Subject selection is to be established by checking through all inclusion/exclusion criteria during screening and prior to enrolment. Deviation from any entry criterion excludes a subject from enrolment into the study. Re-enrolment of subjects in the study is not allowed.

Criteria must be reviewed prior to first treatment to ensure proper eligibility for enrolment. Withdrawal criteria (see section 7.3) must be reviewed at each study visit until last treatment.

7.1 Inclusion Criteria

Subjects meeting the following criteria will be considered for inclusion into the study:

1. Stage I or II BCC or SCC (SCC Well to Moderately Differentiated), and clinically node negative disease
2. Confirmed Histology, and with depth of lesion noted
3. Subjects with up to 3 lesions suitable to enter the study (subjects with more than 3 lesions are not excluded from the study, however 3 target lesions are determined for study evaluation only.)
4. Subjects able and willing to comply with the requirements of the study
5. Age >=18 years
6. Informed Consent signed by the subject consenting to undergo the study
7. Lesions up to 8cm^2
8. Lesions with a depth up to 3mm confirmed on biopsy report AND deemed appropriate clinically by treating clinician
9. Subjects who are not deemed suitable for surgery, for example due to tumour location, performance status or other comorbidities as deemed relevant by the treating clinician
10. Patients who may have declined Surgery and/or fractionated Radiation Therapy

7.2 Exclusion Criteria

A subject will not be eligible for inclusion if any of the following criteria applies during screening or prior to enrolment:

1. Inability to personally provide written informed consent or to understand and collaborate throughout the study
2. Inability or unwillingness to comply with study requirements
3. Prior treatment with surgery or radiation therapy for their target lesion(s)
4. Depth of lesion greater than 3mm as defined by Biopsy and/or clinical assessment
5. Lupus and Scleroderma
6. Basal cell naevus syndrome, xeroderma, vitiligo and albinism
7. Prior laser at the tumour site
8. Malignant melanoma systemic therapy ongoing
9. Any ongoing treatment for malignancy, or in the last 4 weeks prior to study entry
10. A tumour affecting nerves or bony structures
11. Clinical concern of metastatic disease
12. Pregnancy and/or lactation
13. Pathological exclusions: Perineural Invasion, Lymphovascular invasion
14. Anatomical exclusions: NMSC's of the Medial canthus, eyelid margin (upper and lower), Vermillion lip
15. Participation in another clinical trial or administration of any investigational product or experimental product within 60 days prior to screening.

7.3 Withdrawal and Discontinuation

7.3.1 Subject Withdrawal

In general, subjects should not be withdrawn prior to completion of the study.

However, subjects who withdraw consent to participate must be withdrawn from the study. No justification for such a decision is required. If a subject withdraws from the study, the investigator must be informed immediately. If the subject has already received the investigational treatment prior to withdrawal of consent, he/she will be offered to return for a safety follow-up visit. No data obtained after withdrawal of consent will be recorded on case report forms and will not be evaluated as part of the clinical study.

Subjects must be withdrawn if:

- The investigator decides it in the best interest of the subjects, i.e., any situation in which, in the investigator's opinion, a continuation of the treatment with the study medication would be harmful to the subject's safety and well-being.
- The subject is retreated for the same lesion with another therapy or device (other than Rhenium-SCT)

- The subject requires retreatment with Rhenium-SCT

Further reason for withdrawal could be:

- Serious adverse event (SAE) considered to be related to treatment with the investigational medicinal product

In all cases the date, circumstances and any reason provided will be documented on the withdrawal page of the eCRF and in the subject’s medical records.

The sponsor reserves the right to request the withdrawal of a subject due to protocol deviation(s), administrative or any other valid and ethical reason(s).

Subjects withdrawn from the study will be informed about the reasons for their withdrawal.

In any case, if possible, a final study examination should be performed on subjects who discontinue prematurely. This examination should include all procedures performed at the end of treatment visit. All ongoing AEs/SAEs of withdrawn subjects have to be followed up until no more signs and symptoms are verifiable or the subject is in stable condition.

7.3.2 Termination of the Study

The Sponsor reserves the right to prematurely discontinue the study at any time. If the study is terminated or suspended, the sponsor and/or its designees will promptly inform the investigators/institutions and regulatory authorities. The IRB/EC should promptly be informed and provided the reasons(s) for the termination or suspension by the investigator/sponsor, as specified by the applicable regulatory requirement(s).

Reasons for terminating the study may include the following:

- The incidence or severity of AEs in this study indicates a potential health hazard to study subjects
- Subject enrolment is unsatisfactory,
- Data recording is inaccurate or incomplete.

The Sponsor may terminate the study at a study site when the investigator fails to comply with relevant regulations or insufficiently adheres to protocol requirements or recruitment remains insufficient (as deemed so by the sponsor).

8. STUDY TREATMENTS

8.1 Study Treatment

The study treatment under evaluation is Rhenium-SCT.

8.1.1 Treatments Administered

Rhenium-SCT (50Gy dose at target depth) will be administered on Day 0, as a single treatment, by application on a protective foil applied over the area to be treated, such that the radioactive material does not come into contact with the subject. The depth of lesion is determined before treatment by Histology (biopsy).

The lesion is marked out prior to treatment with a 5mm safety margin. The irradiation time required to achieve the desired target dose at the defined penetration depth is calculated based on the amount of radioactivity of the substance being applied, the surface area to be treated and the depth of the lesion. The dose at target depth, the deepest point of the lesion, is defined as 50Gy. This dose, also known as target dose, aims for mortality of tumorous cells at the target depth. After the calculated irradiation time has elapsed, the Rhenium-SCT is removed by pulling the foil from the skin. A full user guide will be provided to the site.

OncoBeta makes a program available to the sites that allows them to verify their calculations. After introducing the input values (lesion size, depth and activity applied), the program will calculate the treatment time needed for each individual lesion, based on the given dose, considered at the deepest point of the lesion (dose at target depth).

This program, which is intended to support the physicists with the patient's dose assessment, is based on VARSKIN 5 calculations, a well-known software used to calculate dose to the skin resulting from exposure to radiation. VARSKIN 5 uses dose point kernels (DPK) that are Monte Carlo based and the code agrees with the EGSnrc Monte Carlo code. User manual will be provided.

Validation of the delivered dose was performed via phantom-based experiments.

The (recommended) dose at target depth used by the software, 50Gy, is based on Dr. Cipriani's work, which is also part of the medical device certification process. 50Gy is the dose approved as part of the CE certification.

The target dose is set to 50Gy. The treatment time will be determined based on the size and depth of the area needed to treat, and the activity applied to it. If a layer is painted thicker on one subject, that would be translated into a higher activity applied, recorded by the dose calibrator, and a reduced treatment time.

8.1.2 Identity of Investigational Products

Rhenium-SCT is obtained from a 188W/188Re generator and has a half-life of about 17 hours, which means that it decays continuously over a short period of time. Rhenium-SCT Molecules produced from the elution of the 188W/188Re generator are bound to a fluid matrix resulting in the compound inside the carpoules. The Rhenium-SCT isotope used for the Rhenium-SCT treatment is a Beta-emitter with main decay energies of maximum 2120 keV with 71% intensity

DocuSign Envelope ID: 61201B2A-687B-46F1-A8DE-FFB5FF22C98F

and 1965 keV with 25% intensity (765 keV on average). In addition to its main Beta-emissions, Rhenium-SCT emits γ-radiation of 155 keV with a 15% intensity, as well as other Beta- and γ-emissions of different energies. Instructions for device preparation can be found in the Rhenium-SCT Technical Dossier.

8.1.3 Packaging and Labelling

Rhenium-SCT Carpoules will be supplied by the Sponsor including a CE compliance certificate, or Therapeutic Goods Administration Australia (TGA) compliance certificate.

Rhenium-SCT Carpoules filled with radioactive compound (medical device part, reference number 0300) will be supplied by the Sponsor, including a CE (Conformitè Europëenne) compliance certificate, or Therapeutic Goods Administration Australia (TGA) compliance certificate. The medical device part will be labelled according to CE mark and ISO14155:2020

The Base Station, Applicator and Measurement Station include the following information in their labelling:

- Serial Number (SN)
- Item Number or Reference Number (REF)
- Year of manufacture
- Manufacturer information

The SN is presented in the form of CCC-NN (CCC = 3-digit batch, NN = 2-digit serial device number within a batch)

For each of the radioactive carpoules, the following information is included in the labelling:

- Serial number
- Date of manufacture
- LOT number
- Activity
- Calibration date and time
- Expiry date
- Position within the transport unit
- Manufacturer information and sponsor information (if applicable)

On the day of the therapy, together with the carpoules, the clinics will receive subject documentation labels with the above-mentioned information for each of the carpoules received (see image of label below).

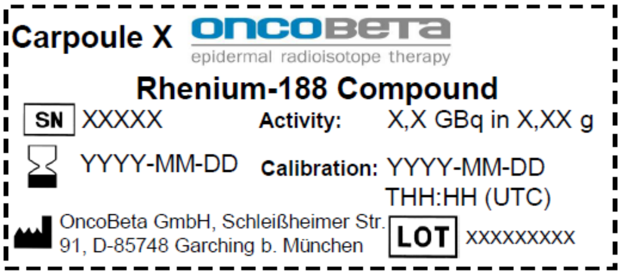

Patient documentation label

Prior to the therapy, the clinics will also receive the Certificate of Compliance (CoC) for the goods, which gives the treating physician a summary of what will be received (template attached):

- LOT/Charge number
- Summary of the carpoules inside the package, with their SN and the activity and mass at the calibration time
- Manufacturer information
- Information about the radionuclide
- Manufacture, calibration and expiry dates

With this document OncoBeta officially confirm that the carpoules received comply with the specification and that we approve its use with patients.

Additional labels accompany the goods, but the ones mentioned above can be used for traceability purposes given that they will be received by the clinics and can be used for their records.

Rhenium-188 compound is filled inside a single-use plastic container, hereinafter referred as carpoules. One carpoule is filled with approx. 300 mg of the rhenium-188 compound and has a maximum activity of 2,2 GBq at the beginning of the treatment. The content of a carpoule is sufficient for the treatment of an area of up to 25 cm^2 and it can be used to treat several lesions and subjects depending on the sizes of the lesions. The therapeutic range of Rhenium-188 beta radiation in unshielded human tissue is about 2-3 mm (92% of the dose is delivered within the first 3 mm).

The carpoules are transported to the treatment centre inside a certified type A package consisting of an inner 35 mm lead container and a big aluminium outer box, considerably reducing the emitting dose. Dose rate limits at the outer surface of the lead container at its maximum loading capacity (around 50 GBq) would be 3 mSv/h and 30 µSv/h at 1 m distance separation. NOTE: the maximum loading capacity is considered at the end of the production before they are shipped to the treatment centre.

8.1.4 Handling and Storage

Details on preparation and administration of the Rhenium-188 compound are provided in the Rhenium-SCT Instructions for use. Rhenium-SCT compound is filled inside the single-use Carpoule, which is the active therapeutic product used to treat subjects (Figure 1). Rhenium-SCT is applied over the area to be treated (tumour or lesion including safety margin) on a protective foil, such that the radioactive material does not come into contact with the subject.

Figure 1. Rhenium-SCT Carpoule

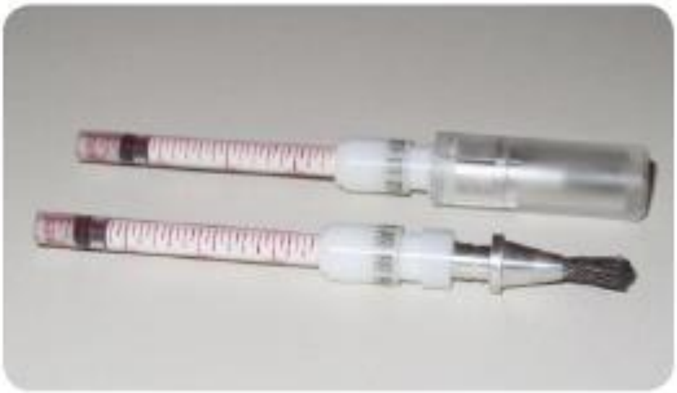

Rhenium-SCT can be applied in any radiation control area with a suitable license for handling Rhenium-188 open sources. The license must cover sufficient annual and daily activity based on the frequency of treatments. A license with annual activity of approximately 500 GBq is recommended for a weekly treatment plan.

Since radiation control areas tend to be limited in space, it is very possible that two or more subjects could be treated simultaneously with the Rhenium-SCT in the same room. The treatment room should have enough treatment beds/chairs for the subjects. Subjects should be placed at least 1.2 m away from each other in order to protect them from cross radiation. Accordingly, the treatment beds/chairs should be positioned in a way that the treated lesions are at least 1.2 m away from the walls of the room, if the material and thickness of the walls are unknown.

The shielding for the room walls must be calculated by a physicist, considering an average specific activity of 100 MBq/cm^2. Due to the weak penetration of the beta emission a minimum of 10 cm concrete wall (outer and inner walls alike) is enough to stop the radiation emitted by the compound. In a worst-case scenario, the radiation burden for a subject due to another subject sitting 1.2m away is negligible and estimated to be around 1.17 µSv. The use of a plastic or lead screen can be used in the unlikely case that two subjects must sit close together and at least one lesion faces the other subject. A few millimetres of plastic (3.5 mm) will drop the dose by >90%.

Treatment will only be conducted at facilities with appropriate radiation licencing.

8.1.5 Compliance and Accountability

The Investigator, designated pharmacist, or radiology personnel will acknowledge receipt of the shipment and note content and condition of the shipment on the clinical material shipping form.

Treatment centres need to have a defined waste management system. Disposals, consisting of empty or unused carpoules, protective foils with Rhenium-188 compound on them and any other radioactive waste, will be disposed of separately over a period of 2-3 weeks for the Rhenium-188 to cool down. The exact waiting time before disposal of the waste depends on the total delivered REC activity and it must be calculated by a physicist. After this period, the

DocuSign Envelope ID: 61201B2A-687B-46F1-A8DE-FFB5FF22C98F

materials can be disposed as non-radioactive waste. If the facility does not have means for the waste management, OncoBeta GmbH can provide special waste containers for this purpose. These waste containers have a 15 mm lead wall and can hold up to 22.5 GBq.

Radiation waste is to be managed as per site Radiation Management Plan.

8.1.6 Prior and Concomitant Therapy

Prior: Other relevant previous medication as judged by the investigator should be documented in the eCRF. Note than subjects who have had surgery and/or radiation therapy for the target lesions are excluded from the study.

Concomitant: concomitant medications should be kept to a minimum during the study. However, if these are considered necessary for the subject’s welfare and are unlikely to interfere with the study objectives, they may be allowed at the discretion of the investigator. Only relevant associated concomitant medications, as deemed appropriate by the investigator, are to be recorded in the eCRF (as deemed appropriate by the investigator).

8.1.7 Unacceptable/Prohibited Therapy

All treatments that the investigator considers necessary for a subject’s welfare may be administered at the discretion of the investigator in keeping with the community standards of medical care.

Subjects may receive other medications that the investigator deems to be medically necessary.

There are no prohibited therapies during the post-treatment follow-up phase. It is however advised that no topical treatments be applied to the treated lesions for approximately 4 weeks following treatment with Rhenium-SCT.

It is left to investigator discretion if subjects are asked to cease therapy with radio sensitising agents before and after Rhenium-SCT treatment. This can be recorded as concomitant medication in the eCRF.

8.1.8 Blinding

This is an open-label study.

9. STUDY PROCEDURES AND ASSESSMENTS

9.1 Study Flow and Visit Schedule

The study-specific assessments and procedures are shown in Table 2.

DocuSign Envelope ID: 61201B2A-687B-46F1-A8DE-FFB5FF22C98F

Table 2. Study Assessments and Procedures Schedule

	Screening day-30	Baseline Day -30 to Day -1	Rhenium-SCT treatment Day 0	post treatment within 14 days	Monthly post 14 day follow up visit for 12 months-Via App	30-day post treatment phone call	3 month follow up	6 month follow up	12 month follow up/ end of study visit	24 months
**Permissible day range**				**+/-48 hours**	**+/-48 hours**	**+/-5 days**	**+/-4 days**	**+/-4 days**	**+/-7 days**	**+/- 14 days**
**Inclusion/ exclusion criteria**	X									
**Urine pregnancy test**			X							
**Histology******	X									
**Informed consent**	X									
**Demographics**		X								
**Medical history**		X								
**Concomitant medications**		X	X	X			X	X		
**Photos via App***					X					
**Photography at site follow up visits (site camera)**		X		X			X	X	X	X
**CTCAE grading/wound assessment**				X			X	X	X	X
**QoL questionnaire* via App**		X						X	X	
**Comfort of treatment* via App**				X						
**Treatment with Rhenium-SCT**			X							
**Cosmetic outcome - Subject rated* via App**									X	X
**Cosmetic outcome - Clinician rated**									X	X
**AEs*****			X	X		X				
**MODIFIED VISUAL RECIST****		X						X	X	X
**Histology if lesion visible/suspected/or per clinical practice**									X	X

DocuSign Envelope ID: 61201B2A-687B-46F1-A8DE-FFB5FF22C98F

**Review for wound complications or radiation changes**										X

*QoL, subject rated cosmetic outcome, monthly photos obtained through subject trial App; ** baseline visual RECIST is to measure lesions only; *** AE's to be recorded for the first 30 days post treatment. After this only treatment related AEs are to be recorded: **** Histology must be confirmed to meet the inclusion criteria before any baseline assessments are conducted

AE: Adverse events; QoL: Quality of life; RECIST: Response evaluation criteria in solid tumours

NOTE: In case of early termination: In case of premature termination during the follow-up period, the investigator shall endeavour to arrange a premature end of study visit and perform examinations according to end of treatment visit. If the subject does not enter the follow-up period, this is the study termination (end of study) visit.

NOTE: Radiation changes at months 24 are to be recorded as the CTCAE grading in protocol appendix 1, excluding Radiation dermatitis.

NOTE: Subjects who do not have a smart phone will complete QoL questionnaire, Comfort of treatment questionnaire and Cosmetic outcome on paper questionnaires at the follow up visits. Monthly photos are not taken for subjects without a smart phone, instead photos are taken at the site, at follow-up visits only.

9.1.1 Informed Consent Visit / Screening and baseline (Day -30 to Day -1)

The subject will be fully informed of all study procedures and implications both verbally and in writing via use of an informed consent form. Once a subject has signed the informed consent form, a subject number (identifier) will be assigned.

Within 30 days prior to the administration of the first dose of study medication, the following procedures and assessments must be completed, and all inclusion and exclusion criteria must be met.

- Obtain informed consent
- Check of inclusion and exclusion criteria, including confirmation of BCC or SCC via histology AND confirmed by clinician review
- Target lesion(s) dimensions including depth of lesion
- Demographic data
- Baseline Modified Visual RECIST (dimensions only)
- QoL Questionnaire (via App or via eCRF or paper for subjects with no phone)
- Medical history
- Relevant concomitant medication
- Photographs of lesions to be treated are taken at the site and uploaded to the eCRF (if a medical photographer is available this is preferable)

The investigator will review all information obtained from the screening procedures. Screening failures, i.e., screened subjects not in compliance with all criteria, are to be excluded and the reason will be recorded. Subjects who fulfil all the inclusion criteria and none of the exclusion criteria will be eligible to participate in the study. Information of subject's study participation can be provided to the subject’s general practitioner upon request, provided the subject has given consent for this to occur and this consent is documented in the source notes accordingly.

9.1.2 Treatment Period

9.1.2.1 Day 0

- On day 0 the subject will receive treatment with Rhenium-SCT for their lesion(s). Any AEs are recorded, as well as relevant concomitant medications.
- Urine pregnancy test (females of child-bearing potential)

9.1.2.2 Day 14

14 days after the subject receives treatment, they are to enter questionnaire responses of their treatment into the subject trial App. For subjects who don’t have a smart phone, this is recorded in the eCRF at the visit using a short questionnaire (see Appendix 4).

Photography is conducted at the site and images loaded to the eCRF. If a medical photographer is available this is favoured, however the site can use an available digital camera.

9.1.2.3 Day 30 Phone call

On day 30, post treatment, the site will call the subject as this forms the end of the AE reported period. After this time, only AEs deemed related to treatment will be collected. During the call any AEs are to be recorded into the eCRF.

9.1.3 Follow-up

Each month following the 14-day site follow-up visit, subjects who have a smart phone will be prompted via the trial App to take 2 photos of each lesion treated (one is taken in indoor light and one in natural light).

Site Follow up visits will occur at 14 days, 3 months, 6 months, 12 months and then 24 months.

<u>3 month follow up visit:</u>

- Concomitant medications
- Photography of treated lesions (medical photographer or site digital camera)
- CTCAE grading (see protocol appendix 1)
- AEs

<u>6 month follow up visit:</u>

- Concomitant medications
- Photography of treated lesions (medical photographer or site digital camera)
- CTCAE grading
- AEs
- QOL questionnaire completed via trial App or on paper during the visit if subject does not have a smart phone
- Modified Visual RECIST assessment

<u>12 month follow up visit:</u>

- Photography of treated lesions (medical photographer or site digital camera)
- CTCAE grading
- Quality of Life questionnaire completed via trial App (or on paper during the visit if subject does not have a smart phone)
- Cosmetic outcome; subject rated. To be completed on Trial app (or directly into the eCRF if the subject does not have a smart phone). VAS image found in the protocol appendix 3
- Cosmetic outcome: Clinician rated. To be completed into the eCRF using VAS in the protocol appendix 3
- Modified Visual RECIST assessment. Guide to assessment is found in protocol appendix 5
- Histology to be performed if lesion is visible, suspected or if this is the site’s regular clinical practice

24 month follow up visit:

- Photography of treated lesions (medical photographer or site digital camera)
- CTCAE grading. Grading can be found in protocol appendix 1
- Cosmetic outcome; subject rated. To be completed on Trial app (or directly into the eCRF if subject does not have a smart phone). VAS image found in the protocol appendix 3
- Cosmetic outcome: Clinician rated. To be completed into the eCRF using VAS in the protocol appendix 3
- Modified Visual RECIST assessment. Guide to assessment is found in protocol appendix 5

- Histology to be performed if lesion is visible, suspected or is the site's regular clinical practice
- General review for wound complications and radiation changes. Radiation changes at months 24 are to be recorded as the CTCAE grading in protocol appendix 1, **excluding** Radiation dermatitis.

9.1.4 Premature Study Termination

In case of premature termination during the follow-up period, the investigator shall endeavour to arrange a premature end of study visit and perform examinations according to end of treatment visit. If the subject does not enter the follow-up period, this is the study termination (end of study) visit.

The last follow up visit (24 months) is the regular study termination visit.

The subject will leave the study if:

- they require alternative or further treatments for their lesions; or
- if they have progressive disease (PD).

9.2 Efficacy Assessments

9.2.1 Response Assessments

The rate of Complete Response (CR) for subjects treated with Rhenium-SCT will be calculated at 12 months post-therapy.

An interim analysis is planned once 50% of the subjects have had their 6-month Follow-Up visit.

Treatment responses will be assessed according to the MODIFIED VISUAL RECIST at Baseline and during the Treatment and Follow-Up phases. If an assessment during the study results in Progressive Disease (PD) according to MODIFIED VISUAL RECIST, subjects will be withdrawn and further treated off study.

9.2.2 Trial App

The trial will have an associated App, available for Apple and Android smart phones, through which the following data will be collected (and will be directly populated into the eCRF):

- QoL questionnaire prior to treatment: (anytime during the screening period, after consent has been given and the subject has been enrolled into the study) and at the 6-, 12- and 24-month Follow-Up visits
- Comfort of Treatment: recorded within 14 days post-therapy with Rhenium-SCT
- Subject rated Cosmetic Outcome: recorded at 12 months and then at 24 months
- Monthly photos for general visual assessment from the 14-day post-therapy visit onwards

The app will be made available in English and German, the language can be selected and changed within the app itself.

The App is available for subjects who have a smart phone with a camera function. **Subjects who <u>do not</u> have a smart phone can still enter the study but will need to complete assessments as follows:**

- Instead of the subject taking photos on a smart phone App monthly, the site staff will take photos at the Follow-up visits and upload the images into the eCRF
- Comfort of treatment will be recorded by the subject via short paper questionnaire at the Day 14 post-therapy visit - and then entered by site staff into the eCRF
- QOL is to be completed by the subject on paper questionnaire and then entered into the eCRF by the site staff at the Follow-up visits. This will be done during the Baseline visit, then at the 6 and 12 month Follow-up visits
- Cosmetic Outcome - subject rated. This is to be entered into the eCRF. The subject will verbalise the response having seen a copy of the VAS scale (in protocol appendix 3) This will be completed at the 12- and 24-month Follow-up visits

Upon enrolment into the study, and once informed consent has been gained, the eCRF will generate a unique App code for the subject.

The subject will download the trial App from the Apple Store or Google Play and enter this unique code in order to access the App and its functionality.

No data recorded via the App is stored on the phone for privacy compliance. All data is immediately transferred from the App to the eCRF or a secure server.

Photos taken by the subject via the Trial App should be:

- The subject may need the assistance of a family member or friend to take the photos if the target lesions are difficult to reach.
- Two separate photos of each target lesion are to be taken: one in indoor light, and one in natural light. The App will prompt the subject accordingly.

The App will deliver pop-up notifications to the subject's smart phone at timepoints where subject reporting is required.

Photos are to be evaluated by the principal investigator (PI) or allocated study nurse. No scoring or assessment is required from the App photos but are to be utilised for general assessment. At the end of study, the App photos may be utilised to assess the lesion over time, in general terms.

At sites where a medical photographer or photography equipment is available, the photos can be taken in this way at Follow-up visits and uploaded directly to the eCRF.

9.2.3 Subject Reported Outcome Measures (PROMs)

Via the trial App (or at follow-up visits for subjects with no smart phone), the following will be recorded:

- QoL questionnaires
- Subject reported Cosmetic Outcome
- Comfort of Treatment

  In addition:
- Monthly photos of treated lesions are taken for general assessment by the site

9.3 Demographics and Medical History

Demographic data to be collected for this study include:

- Gender
- Age
- Ethnicity
- Fitzpatrick score (Fitzpatrick Classification Scale for Skin Types)

The medical history comprises:

- All relevant medical conditions/illnesses in the past and present at the start of the study.

9.4 Safety Assessments

9.4.1 Clinical Laboratory Evaluation

No clinical laboratory evaluations are planned for the study (Rhenium-SCT is administered by application on a protective foil applied over the area to be treated, such that the radioactive material does not come into contact with the subject).

It is noted that histology is to be taken during the screening period to confirm type of lesion as well as depth of lesion.

9.4.2 Pregnancy Test

At Day 0 (treatment day with Rhenium-SCT), urine samples will be analysed for Human Chorionic Gonadotropin (hCG) to assess pregnancy status in females of childbearing potential. All women, including those with tubal ligation, are considered to be of childbearing potential unless they have been postmenopausal for at least 2 years. Hysterectomized women are considered surgically sterile.

9.4.3 Pharmacokinetics

No pharmacokinetic evaluations are planned (Rhenium-SCT is administered by application on a protective foil applied over the area to be treated, such that the radioactive material does not come into contact with the subject).

9.4.4 Pharmacodynamics

No pharmacodynamic evaluations are planned (Rhenium-SCT is administered by application on a protective foil applied over the area to be treated, such that the radioactive material does not come into contact with the subject).

9.4.5 Pharmacogenetics

No pharmacogenetic evaluations are planned (Rhenium-SCT is administered by application on a protective foil applied over the area to be treated, such that the radioactive material does not come into contact with the subject).

10. PHARMACOVIGILANCE

10.1 Definition of Adverse Events

An AE is defined as any untoward medical occurrence in a subject administered a treatment and which does not necessarily have a causal relationship with this treatment. An AE can therefore be any new sign, symptom, illness, or syndrome, any abnormal laboratory values, if judged clinically significant in the opinion of the investigator, any worsening (change in nature, severity or frequency) of a concomitant or pre-existing illness, any adverse effect of the investigational medicinal product/device, including comparator or concomitant medication, any product/device interactions, any adverse effect of an invasive procedure required by the protocol or any accident or injury, whether or not related to the investigational medicinal product/device.

All AEs fall into one of two categories: “non-serious” and “serious”.

In this study, the following medical events will not be considered to fall under the definition of an AE:

- Surgical procedures or other therapeutic interventions themselves are not AEs, but the condition for which the surgery/intervention is required is an AE and should be documented accordingly.
- Planned surgical measures and the condition(s) leading to these measures are not AEs, if the condition(s) was (were) known before the period of observation and did not worsen during study. In the latter case, the condition should be reported as medical history.
- Any SAEs detected by the investigator in a study subject after the end of the period of observation, which the investigator does not consider to be related to prior study treatment or procedures

Progression of the cancer under study is not considered an AE unless it results in hospitalization or death.

The study will collect all AEs for the first 30 days of the study, after which only AE’s deemed treatment (device) related will be collected.

10.2 Definition of Serious Adverse Events (SAEs)

An SAE is any untoward medical occurrence that at any dose (including overdose):

Medical Device (MDR 2017/745)

Serious adverse event is any adverse event that led to any of the following:

a) death,
b) serious deterioration in the health of the subject, that resulted in any of the following:
    1) life-threatening illness or injury,
    2) permanent impairment of a body structure or a body function,
    3) hospitalization or prolongation of patient hospitalization
    4) medical or surgical intervention to prevent life-threatening illness or injury or permanent impairment to a body structure or a body function,

5) chronic disease
c) fetal distress, fetal death or a congenital physical or mental impairment or birth defect

NOTE 1 Planned hospitalization for a pre-existing condition, or a procedure required by the protocol, without serious deterioration in health, is not considered a serious adverse event.

Any adverse events that do not satisfy these descriptions are defined as being non-serious.

10.3 Definition for Medical Device

10.3.1 Adverse Device Effect (ADE)

Adverse event related to the use of an investigational medical device (ISO 14155:2020).

NOTE 1 This definition includes adverse events resulting from insufficient or inadequate instructions for use, deployment, implantation, installation, or operation, or any malfunction of the investigational medical device.

NOTE 2 This definition includes any event resulting from use error or from intentional misuse of the investigational medical device.

10.3.2 Device Deficiency (DD)

An inadequacy of a medical device with respect to its identity, quality, durability, reliability, usability, safety or performance (ISO 14155:2020).

NOTE 1 Device deficiencies include malfunctions, use errors, and inadequacy in the information supplied by the manufacturer including labelling.

NOTE 2 This definition includes device deficiencies related to the investigational medical device or the comparator.

10.3.3 Incident and Serious Incident

Incident means any malfunction or deterioration in the characteristics or performance of a device made available on the market, including use-error due to ergonomic features, as well as any inadequacy in the information supplied by the manufacturer and any undesirable side-effect (MDR 2017/745).

Serious incident means any incident that directly or indirectly led, might have led or might lead to any of the following:

a) the death of a patient, user or other person,
b) the temporary or permanent serious deterioration of a patient's, user's or other person's state of health,
c) a serious public health threat (MDR 2017/745).

10.3.4 Unanticipated Serious Adverse Device Effect (USADE)

A SADE which by its nature, incidence, severity or outcome has not been identified in the current version of the risk analysis report (ISO 14155:2020).

NOTE Anticipated serious adverse device effect (ASADE) is an effect which by its nature, incidence, severity or outcome has been identified in the risk analysis report.

10.4 Period of Observation

All AEs that occur after the consent form is signed but before treatment with investigational medicinal products/devices must be reported by the investigator if they cause the subject to be excluded from the study or are the result of a protocol-specified intervention.

For the purposes of this study, the period of observation for collection of AEs extends from the time when the subject signed informed consent until 30 days after the treatment administration.

If the Investigator detects a SAE in a study subject after the end of the period of observation (i.e., 30 days post treatment to 24 months) and considers the event related to prior study treatment or procedures, he or she should contact the Medical Monitor to determine how the AE should be documented and reported. An SAE form should be completed and sent to Medical Monitor.

Association of an event with the device can be excluded (at investigator discretion if deemed unrelated to treatment) if the event occurs later than the following latency period after the last product/device application:

- 30 days for non-serious adverse events,
- 30 days for SAEs
- 30 days for pregnancy, or 30 days if the subject initiates new anticancer therapy, whichever is earlier

If events occur after these latency periods, they will not be recorded, unless deemed associated (related) with the device treatment.

Any SAE brought to the attention of an investigator at any time outside of the time period specified above must be reported immediately to the Sponsor if the event is considered to be product/device related.

10.5 Documentation and Reporting of Adverse Events

Adverse events, SAEs, and other reportable safety events will be reported by the participant (or, when appropriate, by a caregiver, surrogate, or the participant's legally authorized representative).

The investigator, who is a qualified physician, and any designees are responsible for detecting, assessing, documenting, and reporting events that meet the definition of an AE or SAE as well as other reportable safety events. Investigators remain responsible for following up AE, SAEs and other reportable safety events for outcome.

All AEs (whether serious or non-serious) for the first 30 of the study must be documented in the eCRF. After 30 days, only AE’s and SAE’s deemed treatment associated will be collected.

The Investigator must document all AEs that occur during the first 30 days of the study on the respective pages provided in the eCRF in accordance with the instructions for the completion

of adverse event reports in clinical studies. These instructions are provided in the Investigator file and in the eCRF itself.

Every attempt should be made to describe all AEs in terms of a diagnosis. If a clear diagnosis has been made, individual signs and symptoms should not be recorded unless they represent atypical or extreme manifestations of the diagnosis, in which case they should be reported as separate events. If a clear diagnosis cannot be established, each sign and symptom must be recorded individually.

10.5.1 Nature

The **nature** of the event will be described in precise, standard medical terminology (i.e. not necessarily the exact words used by the subject). If known, a specific diagnosis should be stated (e.g., flu like symptoms).

10.5.2 Severity

The **severity** of the AE will be described in terms of Grades according to according to the CTCAE v.5.0 and according to the investigator’s clinical judgment.

Grade		Definition
Grade 1	mild	asymptomatic or mild symptoms; clinical or diagnostic observations only; intervention not indicated.
Grade 2	moderate	minimal, local or noninvasive intervention indicated; limiting age-appropriate instrumental activities of daily life (ADL)[1]
Grade 3	severe or medically significant but not immediately life-threatening	hospitalization or prolongation of hospitalization indicated; disabling; limiting self-care ADL[2]
Grade 4	life-threatening consequences	urgent intervention indicated
Grade 5	death related to AE	-

10.5.3 Duration

The **duration** of the event will be described by the start date and end date.

For SAEs, the start date is the date on which the event became serious. The end date of an SAE is the date when the event resolved (not when serious criteria were no longer fulfilled).

10.5.4 Relationship

Adverse events are also classified according to the degree of responsibility of the investigational device itself or of the procedure for implementation of a medical device in accordance with the following categories:

- <u>Not related:</u> relationship to the device or procedures can be excluded when:
  - the event has no temporal relationship with the use of the investigational device, or the procedures related to application of the investigational device;
  - the serious adverse event does not follow a known response pattern to the medical device (if the response pattern is previously known) and is biologically implausible;

- the discontinuation of medical device application or the reduction of the level of activation/exposure – when clinically feasible – and reintroduction of its use (or increase of the level of activation/exposure), do not impact on the serious adverse event;
- the event involves a body-site or an organ that cannot be affected by the device or procedure;
- the serious adverse event can be attributed to another cause (e.g. an underlying or concurrent illness/ clinical condition, an effect of another device, drug, treatment or other risk factors);
- the event does not depend on a false result given by the investigational device used for diagnosis, when applicable;

To establish the non-relatedness, not all the criteria listed above might be met at the same time, depending on the type of device/procedures and the serious adverse event.

- Possible: the relationship with the use of the investigational device or comparator, or the relationship with procedures, is weak but cannot be ruled out completely. Alternative causes are also possible (e.g., an underlying or concurrent illness/ clinical condition or/and an effect of another device, drug or treatment). Cases where relatedness cannot be assessed, or no information has been obtained should also be classified as possible
- Probable: the relationship with the use of the investigational device or comparator, or the relationship with procedures, seems relevant and/or the event cannot be reasonably explained by another cause.
- Causal relationship: the serious adverse event is associated with the investigational device, comparator or with procedures beyond reasonable doubt when:
  - the event is a known side effect of the product category the device belongs to or of similar devices and procedures;
  - the event has a temporal relationship with investigational device use/application or procedures;
  - the event involves a body-site or organ that
    - the investigational device or procedures are applied to;
    - the investigational device or procedures have an effect on;
  - the serious adverse event follows a known response pattern to the medical device (if the response pattern is previously known);
  - the discontinuation of medical device application (or reduction of the level of activation/exposure) and reintroduction of its use (or increase of the level of activation/exposure), impact on the serious adverse event (when clinically feasible);
  - other possible causes (e.g., an underlying or concurrent illness/ clinical condition or/and an effect of another device, drug or treatment) have been adequately ruled out;
  - harm to the subject is due to error in use;
  - the event depends on a false result given by the investigational device used for diagnosis, when applicable;

  To establish the relatedness, not all the criteria listed above might be met at the same time, depending on the type of device/procedures and the serious adverse event.

10.5.5 Outcome

The **outcome** of the event will be described in terms of:

- Recovered: fully recovered or by medical or surgical treatment the condition has returned to the level observed at the first study related activity after the subject signed the informed consent

- Recovering: the condition is improving, and the subject is expected to recover from the event. This term should only be used when the subject has completed the study
- Recovered with sequelae: as a result of the AE, the subject suffered persistent and significant disability/incapacity (e.g., became blind, deaf, paralyzed). Any AE recovered with sequelae should be rated as an SAE
- Not recovered
- Fatal
- Unknown: This term should only be used in cases where the subject is lost to follow-up.

Insofar as possible all adverse events should be followed-up to determine the final outcome of the event. Details of follow-up should be recorded on the SAE report form, if applicable (e.g., discontinuation of study medication, if specific treatment is required, if hospitalization is required etc.).

10.6 Documentation and Reporting of Serious Adverse Events

If the AE is serious, the Investigator must complete in addition to the “Adverse Event” eCRF page a “SAE report form” (electronic copy can be downloaded and/printed from the eCRF) at the time the SAE is detected. This form must be sent immediately, i.e., within 24 hours upon becoming aware of the SAE to the Sponsor or delegate by e mail to:

safety@molecule2.com.au

The initial report should be as complete as possible, including details of the current illness and (serious) AE, the reason why the event was considered serious, date of onset and stop date (if applicable), diagnostic procedures and treatment of the event, relevant medical history and concomitant medication and action taken with study medication.

Every attempt should be made to describe all AEs in terms of a diagnosis. If a clear diagnosis has been made, individual signs and symptoms should not be recorded unless they represent atypical or extreme manifestations of the diagnosis, in which case they should be reported as separate events. If a clear diagnosis cannot be established, each sign and symptom must be recorded individually.

For the time period beginning at treatment allocation through 90 days following cessation of treatment, or 30 days following cessation of treatment if the subject initiates new anticancer therapy, whichever is earlier, any SAE, or follow up to a SAE, including death due to any cause other than progression of the cancer under study, whether or not related to the Sponsor's product, must be reported.

Any SAE brought to the attention of an investigator at any time outside of the time period specified above must be reported immediately to the Sponsor if the event is considered to be treatment related.

10.7 Reporting of Pregnancy and Lactation to the Sponsor

Although pregnancy and lactation are not considered adverse events, it is the responsibility of investigators or their designees to report any pregnancy or lactation in a subject (spontaneously reported to them) that occurs during the first 30 days of the study.

Pregnancies that occur after the consent form is signed but before treatment with Rhenium-SCT must be reported by the investigator if they cause the subject to be excluded from the study.

Pregnancies that occur from the time of treatment through 30 days following treatment, must be reported by the investigator.

Follow-up of Missing Information

Information not available at the time of the initial report (e.g., an end date for the AE or laboratory values received after the report) must be documented on a “Serious Adverse Event” form, with the box “Follow-up” checked under “Report type”.

All subjects who have an AE, whether considered associated with the use of the investigational products or not, must be monitored to determine the outcome. The clinical course of the AE will be followed up according to accepted standards of medical practice, even after the end of the period of observation, until a satisfactory explanation is found, or the Investigator considers it medically justifiable to terminate follow-up.

Should the AE result in death, a full pathologist’s report should be supplied, if possible.

The sponsor will identify missing information for each SAE report. Requests for follow up will be sent to the site monitor for further processing. The site monitor will follow up information in regular intervals from the Investigators until all queries are resolved or no further information can be reasonably expected. All responses to queries and supply of additional information by the Investigator should follow the same reporting route and timelines as the initial report.

10.8 Deaths

In the case of a subject dying, the investigator will document the cause of death in the Death CRF page. If possible, a death certificate should be obtained with the cause of death evaluated and documented.

10.9 Expedited Reporting of Adverse Events

10.9.1 Development Safety Update Reports

The sponsor or sponsor representative will prepare and submit development update safety reports (DSURs) for Rhenium-SCT to IRB/IECs as required.

10.9.2 Medical Device Vigilance Reporting

The sponsor evaluates in close collaboration with the investigator, whether the serious adverse event requires vigilance reporting or documentation only according to the applicable

regulations (MDR 2017/745 Art. 87). Vigilance reporting is necessary when the serious incident or field safety corrective action (FSCA) involving device made available on the market.

The sponsor or its representative will take responsibility for notifying (serious) incidents and FSCA immediately to the Competent Authority and the Ethics Committee as applicable per local regulations and timelines.

The sponsor is responsible for reporting periodic safety update reports (PSUR), trend reports, premature termination or suspension of the clinical trial, and the final Study Report to Regulatory Authorities, Ethic Committees and investigators as applicable per local regulations and timelines.

11. STATISTICAL METHODS

11.1 Determination of Sample Size

The primary objective of the study is to estimate CR rate and show non-inferiority to historical values for CR rate following surgery and or radiotherapy. Complete response rate for BCC is 91% at 5 years[1] and for SCC 79%[14]. Review of the published papers for brachytherapy shows the split between BCC and SCC is 2:1[3], 1.68:1[4] and 2.1:1[5].

Based on previous studies, the CR rate for BCC and SCC is expected to be close to 100% following Rhenium-188 SCT treatment. For simplicity, assuming 1 lesion per subject, a sample size of 120 subjects is sufficient to provide at least 80% power to conclude non-inferiority using a one-sided alpha of 0.025 under the following circumstances (Table 3).

Table 3. Sample size calculation

Non-inferiority margin	Assumed CR rate
85%	≥94%
86%	≥94%
87%	≥95%
88%	≥96%
89%	≥97%
90%	≥97%

CR: complete response

Note. A series of simulations (1000) were run to explore the potential impact of different ratios of BCC:SCC on the estimated overall, historical CR rate, assuming the SCC rate was 79% and the BCC rate 91%.

For ratios between 1.5:1 (BCC: SCC) to 3.5:1, the range of median (from simulation) overall CR rate was 87% to 89%. Accordingly, the table above is applicable irrespective of the BCC:SCC ratio.

11.2 Statistical and Analytical Plans

A Statistical Analysis plan will be written for the study.

11.3 Summary of Variables

Continuous variables (e.g., age) will be summarised using descriptive statistics (number of observations, mean, median, minimum, maximum and standard deviation). Categorical

DocuSign Envelope ID: 61201B2A-687B-46F1-A8DE-FFB5FF22C98F

variables (such as gender) will be summarised using frequency tables (presenting the number and percentage). All summary tables will clearly indicate the number of subjects included in the summary.

11.4 Data Sets to be Analysed

11.4.1 Safety Analysis Set

The safety analysis set comprises all subjects who have received treatment and for whom at least one item of safety data is available. Documentation that the subject did not experience any adverse events constitutes safety information.

11.4.2 Full Analysis Set

The full analysis set (FAS) comprises all subjects who provided consent and were enrolled in the study.

Intent to Treat Set

The intent to treat (ITT) set comprises all subjects in the FAS who received treatment and for whom at least one item of efficacy data is available.

11.4.3 Per-Protocol Set

No per protocol set will be defined.

11.5 Summary of Subject Disposition

The number and percentage of subjects in each analysis set will be summarised. In addition, the number (and percentage) of subjects completing the study will be presented. For subjects not completing the study the reason for premature withdrawal will be summarised.

11.6 Baseline and Background Characteristics

All baseline characteristics (demography, medical history) will be summarised using descriptive statistics (mean, median, standard deviation, minimum and maximum) for continuous variables and frequency tables (frequency and percentage) for categorical variables.

11.7 Efficacy Analyses

Primary efficacy endpoint: Complete Remission (CR)

The number of lesions treated per subject will be summarised.

The number (and percentage) of lesions in each category (CR, partial response [PR], stable disease [SD] and progressive disease [PD]) at each timepoint, and the overall best response will be presented. The percentage of lesions achieving CR during the study (i.e., CR is the

best overall response) will be estimated and presented with exact 95% confidence limits overall and by tumour type.

Secondary efficacy endpoints: Change from baseline in QoL, comfort of treatment, and cosmetic outcomes

The QoL questionnaire will be scored according to the author's instructions. Change from baseline in score will be calculated. Descriptive statistics will be presented for actual score and change from baseline by timepoint, overall and by tumour type (BCC/SCC). A mixed model will be fitted with change from baseline as the outcome variable. Baseline score will be included as a covariate and timepoint (6 months/12 months) tumour type, tumour stage as factors. Other relevant prognostic factors assessed at baseline will be included. Subject will be included as a repeated term to acknowledge the data structure. From the model the adjusted mean change at each timepoint will be obtained and presented with 95% confidence limits. Adjusted mean change will be estimated overall and by tumour type.

Comfort with treatment is being assessed using a short questionnaire. The frequency (and percentage) of subjects reporting each option for ach question will be presented.

Cosmetic outcome (subject and clinician) is being assessed at two timepoints (12 months and 24 months respectively) using a Visual Analogue Scale (VAS). Descriptive statistics will be presented overall and by tumour type (BCC or SCC). A mixed model will be fitted with VAS as the outcome variable. Tumour type and tumour stage will be included as factors. Other relevant prognostic factors assessed at Baseline will be included. From the model the adjusted mean score will be obtained and presented with 95% confidence limits. Adjusted mean score will be estimated overall and by tumour type.

11.8 Safety Analyses

11.8.1.1 Adverse Events

Adverse events will be coded using MedDRA and comprehensively summarised by System Organ Class (SOC) and Preferred Term (PT) overall, by severity and by relationship to Rhenium-SCT.

11.8.2 Clinical Laboratory

No clinical laboratory data are being recorded.

11.8.3 Physical Examination and Other Safety Measures

No safety assessments are being performed

11.9 Subgroup Analyses

All analysis models will include tumour type (BCC/SCC) as a factor. As described above, for all analyses, the results will be presented overall and by tumour type

DocuSign Envelope ID: 61201B2A-687B-46F1-A8DE-FFB5FF22C98F

11.10 Interim Analyses

An interim analysis is planned once 50% of subjects have completed 6 month follow up visit.

The purpose of the interim analysis is to provide an early indication of the effects of treatment on patient reported outcomes (namely QOL and subject report comfort of treatment). No amendments to the study are planned on the basis of the interim analysis results. The results may be published or presented at a conference.

Subject recruitment will not be paused for the interim analysis.

11.11 Handling of Dropouts and Missing Data

The reason for withdrawal will be summarised. For all efficacy assessments, all available data will be used. No imputation for missing data will be implemented.

Modified Visual RECIST assessment

- Histology to be performed if lesion is visible, suspected or is site clinical practice

12. DATA COLLECTION, HANDLING, AND RECORD KEEPING

12.1 Generation of Data Base

The eCRF will be supplied by the CRO and should be handled in accordance with the instructions provided. eCRFs will be provided as regulatory compliant, electronically secure and protected, web-based database. An audit trail will record all entries and corresponding changes.

All eCRFs should be filled out completely by qualified personnel or authorized study staff. Only authorized persons will be granted access. After the last query for a subject is closed, the eCRF is reviewed by the investigator and signed electronically.

12.2 Data Collection

All study data are to be recorded in the eCRF.

All the subject's data have to be reported on the eCRF in a pseudonymized fashion, the subject only being identified by the subject number.

The investigator will be responsible for the completeness, accuracy and legibility of the information in the eCRF and other study documents. Data derived from source documents are to be consistent with these source documents.

The study monitors check the eCRF against the source documents for accuracy and validity according to the monitoring schedule, as applicable.

Upon completion of the examination, eCRF completion is expected at each site within 5 days of the subject visit, to ensure quality of data and subject safety. Once eCRF are completed, they will be available for review by the monitor and the clinical data management. Completed eCRF will be reviewed remotely for logical discrepancies. The monitor will ensure that all data queries and subsequent amendments in the eCRF documentation are made according to GCP guidelines.

The trial will have an associated App, available for Apple and Android smart phones, through which the following data will be collected (and will be directly populated into the eCRF):

- QOL questionnaire prior to treatment, anytime during the screening period, after consent has been given, and the subject has been enrolled into the study
- Comfort of treatment, recorded within 14 days post-treatment with Rhenium-SCT
- Subject rated Cosmetic Outcome recoded at 12 months and 24 months post-treatment
- Monthly photos for general visual assessment

If the subject does not have a smart phone, then monthly photos are not taken and all other PROM data is collected at follow visits and then entered into the eCRF. Questionnaires and VAS can be found in the protocol appendix 3.

Upon enrolment into the study, and once the subject's informed consent has been obtained, the eCRF will generate a unique App code for the subject.

The subject will download the trial App from the Apple Store or Google Play and enter this unique code in order to access the App and its functionality.

No data recorded via the App is stored on the phone for privacy compliance. All data is immediately transferred from the App to the eCRF or a secure server.

Photos taken by the subject via the Trial App should be:

- Taken with a ruler next to each lesion. The ruler will be supplied by the site. The subject may need the assistance of a family member or friend to take the photos if the target lesion is difficult to reach.
- Two separate photos of each target lesion are to be taken: one in indoor light, and one in natural light. The App will prompt the subject accordingly.

The app will pop up notifications at timepoints at which subject reporting is required.

12.2.1 Source Data

Source data is all information, original records of clinical findings, observations, or other activities in a clinical study necessary for the reconstruction and evaluation of the study. Source data are contained in source documents. Examples of these original documents and data records include hospital records, physician's and nurse's/technician's notes, clinical and office charts, laboratory notes, pharmacy dispensing records, recorded data from automated instruments, consultant letters, screening and enrolment log, etc.

The investigator is responsible to keep all study-related source data enabling the sponsor to reconstruct the complete course of the clinical study. Source data will be transcribed to and reported through the eCRF accordingly.

12.3 Data Management

All data management activities will be done according to FDA CFR-part 11 as required by regulatory agencies. Responsibility for data management lies with the designated CRO following their internal standard operating procedures (SOPs).

The designated CRO will be responsible for the activities associated with the data management of this study, including the production of an eCRF, setting up a relevant database, along with appropriate validation of data and resolution of queries. All data will be entered into the eCRF. Automated and manual checks will be made against the data to ensure completeness and consistency.

Clean data sets will be provided for statistical analysis and reporting.

12.4 Data Protection

The data obtained during the study will be treated pursuant to the EU General Data Protection Regulation (GDPR) and national laws on data protection.

Generally, all subject related data is recorded in a pseudonymized manner to effectively mitigate the possibility to attribute clinical data to a person in accordance with data protection regulations.

During the clinical study, subjects will be identified solely by means of their individual identification code (Subject ID). Study data stored on a computer will be stored in accordance with local data protection law and will be handled in strictest confidence (e.g., preventing

unauthorised use of the system by password-protection, limited-rights management of authorized personnel, security screening of access to data, access monitoring and an access log of subject data). Distribution of these data to unauthorized persons must be strictly prevented. Data from subjects including imaging analyses, central lab measurements and their results is therefore only accessible to designated personnel on the study and is password-protected. The appropriate regulations of local data legislation will be fulfilled in its entirety.

The subject consents in writing to release the investigator from his/her professional discretion in so far as to allow inspection of original data for monitoring purposes by health authorities and authorized persons (inspectors, clinical monitors, auditors). Authorised persons (inspectors, clinical monitors, auditors) may inspect the subject-related data collected during the study in accordance with the data protection law.

The investigator will maintain a subject identification list (Subject IDs with the corresponding subject names) to enable records to be identified. This list will be stored in the clinical data repository, which is an access-restricted room. Subjects who do not consent to circulate their pseudonymised data will not be included into the study.

This protocol, the CRFs and other study-related documents and material must be handled with strict confidentiality and must not be disclosed to third parties except with the express prior consent of Sponsor. In particular, it must be ensured that the study medication is kept out of reach of third parties. Staffs of the investigators involved in this study are also bound by this agreement.

Electronic data will be handled according to best business practices including, but not limited to, use of appropriate system login details (e.g., for data capturing and management systems), systemic up-to-date virus/malware detection, firewalls, and back-up and restore processes.

In accordance with Oncobeta Therapeutics Australia's SOPs, providers' processes and systems are regularly audited to check their compliance with clinical study related guidelines and regulations and data protection regulations. Reporting procedures include the reporting of breaches as well, enabling Oncobeta Therapeutics Australia to meet such regulatory expectations.

In the unlikely event that un-pseudonymised personal subject data would be received by the sponsor, those would be immediately destroyed by the recipient according to the sponsor's policy.

If the study personnel at the clinical study site becomes aware that a personal data breach has occurred the competent supervisory authority (University Hospital Administration, legal department and law-enforcement institutions) should be notified immediately, i.e., no later than 72 hours after having become aware of it. In the case of the breach is unlikely to result in a risk for the health, rights and freedoms of individuals, the next steps can be discussed and actions to prevent further breaches are installed. The notification of a breach should contain the following key components:

- An explanation of the personal data breach, including the form and extent of the respective data concerned, the name and contact details of the site's Data Controller's Data Protection Officer, an estimation of the possible consequences of the personal data breach.

DocuSign Envelope ID: 61201B2A-687B-46F1-A8DE-FFB5FF22C98F

- In addition, a short description of the measures taken by the controller to address the personal data breach, including, where appropriate, to mitigate its possible adverse effects.
- Subjects are to be notified if the personal data breach is likely to result in a high risk to their rights and freedoms, to allow them to take the precautions and enforce legal alterations to protect further damage. Within the scope of this study, no sequencing data or other sensitive high-throughput data is generated. Therefore, the possible effects of a data breach lie within the personal clinical data.
- Specific measures after theft or breaches of personal medical information include the information of the respective authorities, supporting the fast identification of the source of the information breach and support in retrieving the stolen information.

13. QUALITY ASSURANCE

Information about Quality Assurance can be found in the product technical dossier supplied with this protocol.

The following steps in the process shall ensure the final quality of the therapy:

- Quality controls. Quality controls for the dose calibrator shall be performed prior to the therapy to ensure the intended operation of the device
- Certificate of Compliance. Information in the certificate of compliance received by the clinic shall be used as a validation method after the first measurements are taken
- Dose verification. The treatment time calculations can be verified with the OncoBeta dosimetry program
- Equipment inspection. The equipment shall be inspected prior to the therapy to assure its correct operation
- Protection of the users

13.1 Data Monitoring

Monitoring visits will follow monitoring procedures developed by the designated CRO, as regularly as it is necessary during the conduct of the study until the last eCRFs have been completed and all queries have been resolved, in order to comply with GCP/ISO14155:2020 and to ensure acceptability of the study data for international registration purpose.

Regular monitoring visits by representatives of the Sponsor and/or entrusted CRO at the investigator's site prior to the start and during the course of the study will aid in following the study's progress, ensure utmost accuracy of the data and allow for early detection of possible errors. The trial site will be monitored to ensure the quality of the data collected. The objectives of the monitoring procedures are to ensure that the trial subject's safety and rights as a study participant are respected, that accurate, valid, and complete data are collected, and that the trial is conducted in accordance with the trial protocol, the principles of GCP/ISO14155:2020 and local legislation.

The investigator or supervising physician permits sponsor's employees who are supervising the clinical study (e.g., Clinical Research Associates [CRAs]) to regularly examine the case report forms and ask questions about further records relevant to the study. All investigators agree that the CRA will regularly visit each trial site and assure that they receive appropriate support in their activities at the trial site, as agreed in separate contracts with each site.

The CRA will be given access to review subject's medical records (physician's notes, laboratory printouts etc.), while observing the provisions of data protection legislation, to compare the eCRFs with the participants medical records.

The investigator will ensure access for the CRA to all necessary documentation for trial-related monitoring.

Quality control serves to ensure correct employment of the study medication, adherence to the study protocol and the completeness, plausibility and utility of the data entered in the case

report forms. The cooperation between the investigator or his/her colleagues and the CRA will take into account the current status of GCP/ISO14155:2020 requirements.

For quality assurance, the authorities require a direct comparison between the data recorded in the eCRF and the source data compiled by the investigator. For this purpose, the investigators will allow the CRA of the Sponsor and/or the CRO to inspect the source data compiled for this study. According to legal requirements, it is also possible that the Quality Assurance Department of the CRO and of the Sponsor, or a designated contract auditor or a Regulatory Authority, may want to inspect the logistical procedures as well as to scrutinize the data (audit or inspection). The investigator will allow the persons responsible for the audit or the inspection to have access to the source data and documents and will answer any questions.

13.2 Audits and Inspections

The investigator will permit study-related audits, and inspections by the IRB/IEC, the sponsor, and regulatory authorities of all study-related documents (e.g., source documents, regulatory documents, data collection instruments, study data etc.). The investigator will ensure the availability of applicable study-related facilities (e.g., pharmacy, diagnostic laboratory, etc.) for inspections.

Audits and inspections will be performed in accordance with ICH-GCP, EU Directives, ISO 14155:2020, EU Medical Device Regulation and applicable local regulations to ensure that the clinical study is conducted in compliance with the study protocol requirements.

14. ETHICAL AND LEGAL REQUIREMENTS

14.1 Independent Ethics Committee

The study will only start after approval of the study protocol and all relevant documentation by the IRB/IEC of the participating countries in accordance with applicable regulatory and local requirements.

After completion or termination of the study, the results will be submitted to the IRB/IEC. The study is completed with the last visit of last subject.

14.2 Ethical Conduct of the Study

This study will be conducted in compliance with the study protocol, the ethical principles originating in or derived from the Declaration of Helsinki and in compliance with IRB/IEC, informed consent regulations, and International Conference on Harmonization (ICH) Good Clinical Practices (GCP) Guidelines/ISO 14155:2020. In addition, all local legal and regulatory requirements will be followed. Oncobeta Therapeutics Australia will provide appropriate insurance coverage for the subjects, in accordance with legal requirements and local law. An insurance certificate will be provided for the investigator's site file. Subjects are informed by the subject information form, of the existence of such insurance and that they have the right to inspect the terms and conditions of said insurance or may receive a copy, if required per national legislation.

14.3 Changes in the Conduct of the Study

Changes to the protocol during the study will be documented as amendments. The amended protocol will be signed by the relevant personnel at Oncobeta Therapeutics Australia and by the Investigator. Depending on the contents of the amendment and local legal requirements, the amendment will be submitted to the relevant IRB/IEC and, where necessary, to the relevant competent authorities. The Investigator should not implement any deviation from, or changes of the protocol, without agreement by Oncobeta Therapeutics Australia and prior review and documented approval/favourable opinion of the appropriate IRB/IEC and, if legally required, competent authorities, except where necessary to eliminate an immediate hazard to the subjects, or when the change(s) that were approved by Oncobeta Therapeutics Australia involve only logistical or administrative aspects of the study.

The study, and any amendments, will only be implemented following compliance with all legally required regulatory requirements.

14.4 Subject Information and Consent

Generally, interest in clinical studies for non-melanoma skin cancer is high among subjects and subject working groups. Interested subjects will be seen for an initial interview and the general possibility of participation in the study is discussed based on the available reports, imaging studies and lab values. Local hospitals and practitioners will also be involved and send interested subjects for initial interviews.

The Subject Information will be used to explain to the subject the risks and benefits of study participation.

The Consent Sheet will comply with all applicable regulations governing the protection of human subjects, including ICH GCP guidelines, the Declaration of Helsinki, ISO14155:2020 standard, subject confidentiality and data protection. The IEC's written approval of the Subject Information and Consent Sheet will be obtained.

Prior to screening, subjects will be provided with a copy of the approved Subject Information and Consent Sheet. The investigator or an authorized member of the study team will discuss with the subject the nature of the study and the treatments to be administered (including the risks and discomforts to be expected). Subjects will have sufficient opportunity to inquire about details of the study, to discuss study related issues with their families and to decide whether to participate. They will be instructed that they are free to withdraw their participation in the study at any time and for any reason without prejudice.

The investigator will ensure that appropriate signatures and dates on the informed consent document are obtained prior to the performance of any protocol procedures and prior to the administration of study treatments. The investigator will provide each subject with a copy of the signed and dated consent and document the provision of consent in the subject's source notes.

The investigator will inform subjects of new information that may be relevant to the subjects' willingness to continue participation in the study and is also obligated to protect the subjects' confidentiality. Any revision of the Subject Information and Consent Sheet must be approved by the IEC prior to use.

14.5 Confidentiality

The investigators, designated CRO, Oncobeta Therapeutics Australia, and all other involved parties will preserve the confidentiality of all subjects taking part in the study, in accordance with ICH GCP/ISO 14155:2020 and local regulations. The confidentiality of all subject identities will be maintained, except during source data verification, when monitors, auditors and other authorized agents of the sponsor or its designee, the IECs approving this research, as well as any other applicable regulatory authorities will be granted direct access to the study subjects' original medical records. No material bearing a subject's name will be kept on file by designated CRO or Oncobeta Therapeutics Australia. The data retained from this study will be protected in accordance with all applicable legal requirements.

Information about study subjects will be kept confidential and managed according to the EU Directive 2001/20/EC, 2005/28/EC, 2003/63/EC and EU Regulation No 536/2014 and relevant national and local legislations.

14.6 Finance, Subject Insurance Coverage and Investigator Indemnity

Financing agreements will be managed by the sponsor in a separate document.

Oncobeta Therapeutics Australia will provide appropriate insurance coverage for the subjects, in accordance with legal requirements and local law. An insurance certificate will be provided for the Investigator's Site File. Subjects are to be informed by the investigator of the existence of such insurance and that they have the right to inspect the terms and conditions of said insurance or may receive a copy, if required per national legislation.

14.7 Publication of Study Results

A final clinical study report will be prepared according to the ICH guideline on 'Structure and Content of Clinical Study Reports' (ICH E3). A final clinical study report will be prepared regardless of whether the study is completed or prematurely terminated. The clinical study report will be the sole property of the sponsor. Publication of the clinical study report or of parts of it may only be allowed when authorized by the sponsor. Reports to the authorities and IEC after study termination will be provided and results will be posted in the Clinical Study Register as required by law.

The investigator and the institution recognize that all data generated from this study are the proprietary and confidential information of the sponsor, along with all information supplied by the sponsor. The sponsor recognizes the Investigator's and the institution's rights and obligations, as an academic partner, to publish the study results. The study results will be published in accordance with the good publication practice guideline of the international society for medical publication professionals. This includes but is not limited to the following principles:

- The sponsor confirms the authors' freedom to make public or publish the study results and grants full access to the study data for this purpose. The investigator and the institution agree that they are not permitted to publish data related to the study independent of the sponsor and the other investigators.
- The investigator and the institution plan and produce publications in a timely manner and avoid premature release of study information.
- The sponsor and the Investigator and other individuals who have expertise in the area and who are willing to interpret the data and write or review articles and presentations will form a publication steering committee to oversee the preparation of articles and presentations from this study.

Prior to any written or oral presentation of the study results or any part thereof, the investigator or the institution shall send the full text of the proposed disclosure to the sponsor (or, if applicable, the publication steering committee) for review and comments at least 60 business days prior to submission for publication or oral presentation. The sponsor reserves the right to have deleted from the proposed publication any confidential information of the sponsor with the exception for results or data generated on the basis of the study which are of scientific interest to the investigator, and which may be published in compliance with this section. During the above-mentioned period, sponsor in their sole discretion will take the steps he deems necessary to secure any intellectual property arising from the study results or data, including the filing or substantiating of one or more patent applications. The investigator or the institution shall follow customary principles related to scientific publications in determining and attributing authorship of any proposed publication, provided that any such publication shall acknowledge the sponsorship by the sponsor.

DocuSign Envelope ID: 61201B2A-687B-46F1-A8DE-FFB5FF22C98F

REFERENCE LIST

1. Wilder, R.B., Shimm, D.S., Kittelson, J.M., Rogoff, E.E., and Cassady, J.R. (1991). Recurrent basal cell carcinoma treated with radiation therapy. Arch Dermatol *127*, 1668–1672.

2. Sedda, A., Rossi, G., and Cipriani, C. (2014). DISTRIBUTION IN MICE OF 99M TC(V)-DMSA AND 188 RE(V)-DMSA. undefined.

3. Cipriani, C., Frisch, B., Scheidhauer, K., and Desantis, M. (2017). Personalized High-Dose-Rate Brachytherapy with Non-Sealed Rhenium-188 in Non-Melanoma Skin Cancer. International Journal of Nuclear Medicine Research *Special Issue*.

4. Cipriani, C., Desantis, M., Dahlhoff, G., Brown, S.D., Wendler, T., Olmeda, M., Pietsch, G., and Eberlein, B. (2020). Personalized irradiation therapy for NMSC by rhenium-188 skin cancer therapy: a long-term retrospective study. J Dermatolog Treat, 1–7.

5. Sedda, A., Rossi, G., Cipriani, C., Carrozzo, A.M., and Donati, P. (2008). Dermatological high-dose-rate brachytherapy for the treatment of basal and squamous cell carcinoma. Clin Exp Dermatol *33*, 745–749.

6. Griffin, L.L., Ali, F.R., and Lear, J.T. (2016). Non-melanoma skin cancer. Clinical Medicine *16*, 62–65.

7. Lomas, A., Leonardi-Bee, J., and Bath-Hextall, F. (2012). A systematic review of worldwide incidence of nonmelanoma skin cancer. Br J Dermatol *166*, 1069–1080.

8. Leigh, I.M. (2014). Progress in skin cancer: the U.K. experience. Br J Dermatol *171*, 443–445.

9. Ali, F.R., and Lear, J.T. (2013). Systemic treatments for basal cell carcinoma (BCC): the advent of dermato-oncology in BCC. Br J Dermatol *169*, 53–57.

10. Carrozzo, A.M., Cipriani, C., Donati, P., Muscardin, L., and Sedda, A.F. (2014). Dermo Beta Brachytherapy with 188Re in extramammary Paget's disease. G Ital Dermatol Venereol *149*, 115–121.

11. Castellucci, P., Savoia, F., Farina, A., Lima, G.M., Patrizi, A., Baraldi, C., Zagni, F., Vichi, S., Pettinato, C., Morganti, A.G., et al. (2021). High dose brachytherapy with non sealed 188Re (rhenium) resin in patients with non-melanoma skin cancers (NMSCs): single center preliminary results. Eur J Nucl Med Mol Imaging *48*, 1511–1521.

12. Bhusari, P., Shukla, J., Kumar, M., Vatsa, R., Chhabra, A., Palarwar, K., Rathore, Y., De, D., Kumaran, S., Handa, S., et al. (2017). Noninvasive treatment of keloid using customized Re-188 skin patch. Dermatol Ther *30*.

13. Shukla, J., and Mittal, B.R. Abstract: 188Re Tailor Made Skin Patch for the Treatment of Skin Cancers and Keloid: Overview and Technical Considerations. https://www.cosmosscholars.com/special-issues-ijnmr/46-abstracts/ijnmr/736-abstract-188re-tailor-made-skin-patch-for-the-treatment-of-skin-cancers-and-keloid-overview-and-technical-considerations.

DocuSign Envelope ID: 61201B2A-687B-46F1-A8DE-FFB5FF22C98F

14. Carrozzo, A.M., Sedda, A.F., Muscardin, L., Donati, P., and Cipriani, C. (2013). Dermo beta brachytherapy with 188-Re in squamous cell carcinoma of the penis: a new therapy. Eur J Dermatol *23*, 183–188.

15. Lepareur, N., Lacœuille, F., Bouvry, C., Hindré, F., Garcion, E., Chérel, M., Noiret, N., Garin, E., and Knapp, F.F.R. (2019). Rhenium-188 Labeled Radiopharmaceuticals: Current Clinical Applications in Oncology and Promising Perspectives. Frontiers in Medicine *6*, 132.

16. Mary-Margaret Chren, Rebecca J. Lasek, Linda M. Quinn, Eliot N. Mostow, Stephen J. Zyzanski, Skindex, a Quality-of-Life Measure for Patients with Skin Disease: Reliability, Validity, and Responsiveness, Journal of Investigative Dermatology, Volume 107, Issue 5, 1996,

17. Chren MM, Lasek RJ, Quinn LM, Mostow EN, Zyzanski SJ. Skindex, a quality-of-life measure for patients with skin disease: Reliability, validity, and responsiveness. J Invest Dermatol 1996;107:707-13.

APPENDICES

Appendix 1: CTCAE grading Version 5.0

	Grade 1	Grade 2	Grade 3	Grade 4	Grade 5
**Radiation dermatitis**	Faint erythema or dry desquamation	Moderate to brisk erythema; patchy moist desquamation, mostly confined to skin folds and creases; moderate oedema	Moist desquamation in areas other than skin folds and creases; bleeding induced by minor trauma or abrasion	Life-threatening consequences; skin necrosis or ulceration of full thickness dermis; spontaneous bleeding from involved site; skin graft indicated	Death
**Skin ulceration**	Combined area of ulcers <1 cm; no blanchable erythema of intact skin with associated warmth or oedema	Combined area of ulcers 1 - 2 cm; partial thickness skin loss involving skin or subcutaneous fat	Combined area of ulcers >2 cm; full-thickness skin loss involving damage to or necrosis of subcutaneous tissue that may extend down to fascia	Any size ulcer with extensive destruction, tissue necrosis, or damage to muscle, bone, or supporting structures with or without full thickness skin loss	Death
**Alopecia**	Hair loss of <50% of normal for that individual that is not obvious from a distance but only on close inspection; a different hair style may be required to cover the hair loss, but it does not require a wig or hair piece to camouflage	Hair loss of >=50% normal for that individual that is readily apparent to others; a wig or hair piece is necessary if the patient desires to completely camouflage the hair loss; associated with psychosocial impact	-	-	-
**Skin induration**	Mild induration, able to move skin parallel to plane (sliding) and perpendicular to skin (pinching up)	Moderate induration, able to slide skin, unable to pinch skin, limiting instrumental ADL	Severe induration; unable to slide or pinch skin; limiting joint or orifice movement (e.g., mouth, anus); limiting self-care ADL	Generalized; associated with signs or symptoms of impaired breathing or feeding	Death
**Hypopigmentation**	Hypopigmentation or depigmentation covering <10% BSA; no psychosocial impact	Hypopigmentation or depigmentation covering >10% BSA; associated psychosocial impact	-	-	-
**Hyperpigmentation**	Hyperpigmentation covering <10% BSA; no psychosocial impact	Hyperpigmentation covering >10% BSA; associated psychosocial impact	-	-	-
**Telangiectasia**	Telangiectasias covering <10% BSA	Telangiectasias covering >=10% BSA; associated with psychosocial impact	-	-	-

Appendix 2: QOL Questionnaire

Skin Cancer Index (SCI)

The following questions ask about your views on skin cancer or its treatment and how it may affect you socially, at work, or at home, and other areas of concern. For each of the following, please indicate how much your skin cancer affects your life by marking an "X" in the **one** box that most closely matches how you feel at the present time.

During the past month, how much have you…

	Very Much	Quite a Bit	Moderately	A Little Bit	Not at All
1. Worried that your skin cancer will spread to another part of your body?	☐	☐	☐	☐	☐
2. Felt anxious about your skin cancer?	☐	☐	☐	☐	☐
3. Worried that family members may also develop skin cancer?	☐	☐	☐	☐	☐
4. Worried about the cause of skin cancer?	☐	☐	☐	☐	☐
5. Felt frustrated about your skin cancer?	☐	☐	☐	☐	☐
6. Worried that your tumor may become a more serious type of skin cancer?	☐	☐	☐	☐	☐
7. Worried about new skin cancers occurring in the future?	☐	☐	☐	☐	☐
8. Felt uncomfortable when meeting new people?	☐	☐	☐	☐	☐
9. Felt concerned that your skin cancer may worry friends or family?	☐	☐	☐	☐	☐
10. Worried about the length of time before you can go out in the public?	☐	☐	☐	☐	☐
11. Felt bothered by people's questions related to your skin cancer?	☐	☐	☐	☐	☐
12. Felt embarrassed by your skin cancer?	☐	☐	☐	☐	☐
13. Worried about how large the scar will be?	☐	☐	☐	☐	☐
14. Thought about how skin cancer affects your attractiveness?	☐	☐	☐	☐	☐
15. Thought about how noticeable the scar will be to others?	☐	☐	☐	☐	☐

Appendix 3: VAS Cosmetic outcome (clinician and subject rated)

On a scale of 0-10 (0 being very poor and 10 being no visible wound) what is the cosmetic outcome of the Rhenium-SCT treated lesion(s)?

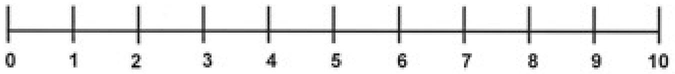

Appendix 4: Comfort of treatment questionnaire

(Completed by subject at day 14 visit or via Trial App on day 14)

We would like to know how you felt whilst receiving your Rhenium treatment. In particular, any pain that may have been felt. Please note that pressure or discomfort do not constitute pain for the purpose of this question. Also please keep in mind if your pain is due to your lesions, rather than the treatment itself.

**Name**	**Menu option circle response**
**Have you ever experienced any pain at your lesion site(s) prior to treatment?**	-Yes<br>-No
**If yes, was your pain present prior or just prior to Rhenium SCT treatment?**	-Yes<br>-No
**Did You feel any pain during your treatment with Rhenium SCT? Please remember this applies to the treatment itself and not pain of your lesion site(s) and pressure is not included as pain.**	-Yes<br>-No
**If yes, at what point during treatment did you experience pain?**	- During the application<br>- During removal of the foil<br>- After the application
**If selected during removal of foil, then please answer:**<br>**was this pain comparable to the removal of an ordinary band aid/plaster?**	-Yes<br>- No

**Name**	**Menu option circle response**
**If selected no:**<br>**How painful was the removal on a scale of 1-5 (1 being minimal pain and 5 being very painful)**	1 2 3 4 5

Appendix 5: Modified Visual RECIST

Modified Visual RECIST Quick Reference:

Eligibility

- Only patients with measurable NMSC lesions at baseline should be included

Measurable disease - the presence of at least one measurable lesion and its neoplastic nature should be confirmed by histology**.**

Measurable lesions - lesions that can be accurately measured in at least one dimension with longest diameter ≥5 mm with the naked eye.

- All measurements should be taken and recorded in metric notation, using a ruler or callipers. All baseline evaluations should be performed as closely as possible to the beginning of treatment and never more than 4 weeks before the beginning of the treatment.
- The same method of assessment and the same technique should be used to characterize each identified and reported lesion at baseline and during follow-up.
- Clinical lesions will only be considered measurable when they are superficial (e.g., skin nodules and palpable lymph nodes). For the case of skin lesions, documentation by colour photography, including a ruler to estimate the size of the lesion, is recommended.

Methods of Measurement

- Visually assess the lesion(s) to measure target lesions selected for response assessment.
- Lesions on contoured surfaces such as the ear are acceptable as measurable lesions.
- Confirm the complete disappearance of lesions assessed by clinical examination.
- The utilization of Dermascopy is permissible as required and should be included as part of the 6 month follow up visit.
- Tumour histology alone cannot be used to assess response. If Histology is taken at the 12-month mark, then this histology must be negative in addition to CR visually, for a patient to be considered in complete response.
- Histology can be used to differentiate between PR and CR at the 12-month mark if tumour is visible, suspected or is site clinical practice to do so.

Baseline documentation of Target lesions

- All measurable lesions up to a maximum of three lesions should be identified as ***target lesions*** and recorded and measured at baseline.
- Target lesions should be selected on the basis of their size (lesions with the longest diameter) and their suitability for accurate repeated measurements (either by imaging techniques or clinically). In addition, the lesions and subject should meet the inclusion criteria.
- A sum of the longest diameter (LD) for *all target lesions* will be calculated and reported as the baseline sum LD. The baseline sum LD will be used as reference by which to characterize the objective tumour.

Response Criteria

Evaluation of target lesions

* Complete Response (CR):	Disappearance of all target lesions
* Partial Response (PR):	At least a 30% decrease in the sum of the LD of target lesions, taking as reference the baseline sum LD
* Progressive Disease (PD):	At least a 20% increase in the sum of the LD of target lesions, taking as reference the smallest sum LD recorded since the treatment started or the appearance of one or more new lesions
* Stable Disease (SD):	Neither sufficient shrinkage to qualify for PR nor sufficient increase to qualify for PD, taking as reference the smallest sum LD since the treatment started

Evaluation of best overall response

The best overall response is the best response recorded from the start of the treatment until the 24 month follow up (taking as reference for PD the smallest measurements recorded since the treatment).

Table 4. Evaluation of best overall response

Target lesions At 12 months	New Lesions at treated site(s)	Overall response at 24 months
CR	No	CR
PR	No	PR
SD	No	SD
PD	Yes or No	PD
CR	Yes	PD

PR	Yes	PD

CR: complete response; PR: partial response; SD: stable disease; PD: progressive disease

- In some circumstances it may be difficult to distinguish residual disease from normal tissue. When the evaluation of complete response depends on this determination, it is recommended that the residual lesion be investigated (biopsy/Histology) to confirm the complete response status.

Duration of stable disease

- SD is measured from the treatment until the criteria for disease progression are met (during the study period), taking as reference the smallest measurements recorded since the treatment started.

Response review

- Response is determined by the site PI

Table 5. Example Modified Visual RECIST data collection:

	Location	Baseline	Follow up 6 months	Follow up 12 months	Follow up 24 months
		Dimensions	Dimensions	Dimensions	
**Target lesion 1**	inner right ear	2.3x2.1	1.8x1.5	1.6x1.3	
**target lesion 2**	outer right ear	2.4x1.2	2.0x0.9	1.5x0.7	
**Target lesion 3**					
**Sum Longest Diameter**		4.7	3.8	3.1	
**Response Rate %**		N/A	19.1	34	
**Response Category**			Stable Disease	Partial Response	

Appendix 6. Guidance notes on Bleeding post treatment

Rhenium SCT is a non-invasive therapy. This poses an advantage over treatments such as surgery or taking medications. This also means that patients can continue other medications

they may be taking. Patients can take their previous medications in the usual way. This also applies to anticoagulants.

Now, due to the treatment itself, the Rhenium SCT has no influence on bleedings, neither directly nor indirectly. Nevertheless, three situations are conceivable during the treatment in which, theoretically, small superficial bleeding can occur.

The first possibility for a bleeding arises, when preparing for intervention. Scabs over the tumour need to be removed and even though this is not necessarily a traumatic procedure, the crusts can stick to the ground of the lesion. Loosening them can cause superficial bleeding.

The second situation where bleeding could theoretically occur, is when removing the foil. Small clots can be torn out by the adhesive film and cause the bleeding.

The third situation where bleeding could happen, is when the necrosis after some weeks is removed. If the crust / necrosis is still adhering to the substrate, abrupt removal of it can trigger local bleeding.

In all cases, purely local measures are required to stop the bleeding. The bleeding site should preferably be compressed mechanically until the bleeding stops. If this is not sufficient, the bleeding can be stopped with the help of fibrin glue.
